# Greater neural overlap between motor imagery and working memory than with movement execution: A meta-analytic comparison

**DOI:** 10.1162/IMAG.a.1095

**Published:** 2026-01-12

**Authors:** Elise E. Van Caenegem, Marcos Moreno-Verdú, Baptiste M. Waltzing, Gautier Hamoline, Frahm Lennart, Robert M. Hardwick

**Affiliations:** Brain, Action, And Skill Laboratory, Institute of Neurosciences, UCLouvain, Belgium; Institute of Neuroscience and Medicine, Brain & Behaviour (INM7), Research Centre Jülich, Jülich, Germany; Department of Psychiatry, Psychotherapy and Psychosomatics, School of Medicine, RWTH Aachen University, Aachen, Germany

**Keywords:** mental imagery, motor imagery, movement execution, working memory, meta-analysis, neuroimaging data

## Abstract

Motor imagery, the mental simulation of movement without physical execution, has traditionally been linked to the neural mechanisms of actual movement, as proposed by Motor Simulation Theory. However, recent frameworks, such as the Motor-Cognitive Model, suggest a closer link between motor imagery and executive functions, particularly working memory. This meta-analytic study quantitatively compared data from 697 neuroimaging studies on motor imagery, movement execution, and working memory. Activation likelihood estimation (ALE) and conjunction analyses revealed that 60% of the volume recruited by motor imagery was also recruited by working memory, while by comparison only 28% of the motor imagery network was also recruited by movement execution. To ensure this difference was not due to variations in overall network sizes, volume-matched analyses were conducted and confirmed a consistent effect but with less dramatic difference (36% of the motor imagery network was also recruited by working memory, compared with 32% for movement execution). Analyses by brain areas indicated that the greater overlap between motor imagery and working memory was primarily due to greater activation in frontal and parietal areas. These findings highlight that motor imagery may place a greater emphasis on cognitive processing than has been assumed by previous models and suggests that central executive processes may play an important role in motor imagery.

## 1 Introduction

Motor imagery can be defined as ”a dynamic state during which a subject mentally simulates a given action” (Decety, 1996). Many studies have looked at the similarities between motor imagery and movement execution, revealing parallels between them at the level of the neural circuits recruited; for example, fMRI studies have demonstrated that motor imagery and actual movement execution share similar brain activations (Sharma & Baron, 2013). Other studies have also shown a similarity in muscle activations during imagery and execution, but with some variations across studies (Guillot et al., 2007). These similarities between motor imagery and movement execution are the subject of the “Motor Simulation Theory”, established by Jeannerod (2001). Motor Simulation Theory argues that “covert actions (imagined movements) are in fact actions, except for the fact that they are not executed”, suggesting that the same neural pathways are recruited during both imagined and physically performed actions. According to Jeannerod (2001), motor imagery involves the programming and generation of movement without its execution, serving as part of a broader process related to intention and preparation for action. As such, Motor Simulation Theory is consistent with theories proposing that the neural mechanisms involved in motor imagery and movement execution are “functionally equivalent” (Crammond, 1997; Grafton et al., 1996; Grèzes & Decety, 2001). Based on a qualitative assessment of studies examining movement execution, motor imagery, and action observation, Jeannerod (2001) suggested that action simulation was supported by a series of motor areas (primary motor, premotor, and supplementary motor cortices, as well as the corticospinal pathway, basal ganglia, and cerebellum) and associative regions (parietal and prefrontal cortices).

A recent intracortical recording study further challenges the notion of strict functional equivalence between motor imagery and execution. Dekleva et al. (2024) showed that activity in the primary motor cortex during imagined wrist movements does not simply reflect attenuated versions of executed dynamics. Instead, population activity was organized into three orthogonal subspaces: one shared between execution and imagery, one unique to execution, and one unique to imagery. Crucially, the imagery-unique subspace preserved the dynamical structure of execution while remaining output-null, thus avoiding overt motor commands. These findings suggest that motor cortex activity during motor imagery is characterized by a reorientation of neural dynamics rather than by a direct equivalence to execution. While Motor Simulation Theory has been widely accepted in the literature, more recent work has challenged the idea that imagined and physically performed actions are functionally equivalent. For example, the literature is inconsistent regarding the direct involvement of the primary motor cortex during action simulation (Riggio & Gangemi, 2024). Studies using Transcranial Magnetic Stimulation (TMS) have applied stimulation over the primary motor cortex and measured the excitability of the corticospinal tract during both motor imagery and control conditions. While studies applying TMS over the primary motor cortex show an increase in corticospinal excitability during motor imagery, these effects may in fact originate from changes in the excitability of premotor regions that contribute to the corticospinal tract (Fadiga et al., 2005). By contrast, other studies using electroencephalography (EEG) show no activation in this region during imagery (Silva et al., 2020), and meta-analytic syntheses of neuroimaging studies indicate that imagery preferentially recruits higher-order associative regions rather than lower-level sensorimotor areas (Van Caenegem et al., 2024). This principle of functional equivalence had been questioned, and new theories emerged (for a comprehensive review see Hurst & Boe, 2022), such as the Motor-Cognitive model (Glover et al., 2020; Glover & Baran, 2017; Martel & Glover, 2023), which asserts that motor imagery and movement execution do not rely on identical mental processes. Specifically, the Motor-Cognitive model proposes that motor imagery and execution share early movement planning mechanisms, but diverge during action implementation, with imagery relying more heavily on executive functions in a fashion similar to working memory, whereas execution depends on sensorimotor circuits and external feedback.

Indeed, while Motor Simulation Theory only relies on similarities between motor imagery and movement execution and does not provide any information about the disparities between them, the Motor-Cognitive model directly addresses these discrepancies. These differences were studied using experiments measuring the timing of real or imagined movements. According to Motor Simulation Theory, the time to imagine a movement should be the same as the time to perform the same movement, as the same neural processes are involved in both situations (Decety & Michel, 1989; Jeannerod, 2001). Glover and Baran (2017) extended this approach by introducing a secondary cognitive task (i.e. mental arithmetic) during motor imagery, which significantly increased imagery duration without affecting execution. This supports the Motor-Cognitive model, highlighting the role of executive resources in motor imagery.

Recent studies have also proposed hybrid models in which motor imagery involves both motor simulation and working memory (Hurst & Boe, 2022). In such models, early planning relies on motor simulation, but later stages of imagery engage working memory and cognitive elaboration, particularly for complex or novel actions. This stage-based perspective generates clear, testable predictions regarding the cognitive demands of imagery versus execution.

Martel and Glover (2023) provided further evidence by using TMS over the dorsolateral prefrontal cortex (DLPFC), showing that disrupting its function prolongs imagery duration, similar to its effect on a calculation task, without impairing movement execution. These results are consistent with the predictions of the Motor-Cognitive model, as selectively interfering with executive resources affects imagery but not actual execution.

Recent work has also challenged the concept of functional equivalence. Hardwick et al. (2018) used a meta-analytic approach to examine the convergence and divergence between brain activity during motor imagery and movement execution. Comparing the brain networks recruited during motor imagery and movement execution, they identified significant overlap in the sensorimotor and subcortical areas. These findings indicate that imagining an action engages brain areas similar to those involved in action preparation. However, the majority of the voxels identified were not commonly activated between motor imagery and movement execution; particularly, the dorsolateral prefrontal cortex was only consistently recruited during motor imagery, and not during movement execution. Similarly, there were notable differences in the overall extent and sub-regions involved in each task. For example, motor imagery and movement execution showed similar activations in premotor and parietal regions, but at the level of subcortical regions such as the putamen and the cerebellum, movement execution activates these regions to a greater extent compared with motor imagery. These data suggest that while there is indeed some overlap between motor imagery and movement execution, the principle of functional equivalence does not fully explain the available data, as there were notable discrepancies between the networks for each task.

The emergence of the Motor-Cognitive model has led to further examination of the link between motor imagery and executive function, and more specifically working memory. Also known as updating, working memory tasks involve storing and performing operations with task-related information in short-term memory, recalling, or recognizing it later, and modifying the information based on the task’s context, which may include manipulating the information as part of the task (A. Baddeley, 2012; A. D. Baddeley & Hitch, 1994). Glover and Baran (2017) argue that the elaboration and monitoring of motor imagery in real time rely on a central pool of executive resources, similar to their use in working memory. As both working memory and motor imagery involve maintaining and manipulating task-relevant information, there is reason to consider that they may recruit similar brain networks.

The present study, therefore, examined the neural networks recruited during movement execution, motor imagery, and working memory. Our aim was to examine the specific neural network for each paradigm (movement execution, motor imagery, and working memory), in order to determine the extent to which the network recruited during motor imagery was also recruited during these other paradigms. This process aimed to address key limitations in current literature; specifically, while Jeannerod (2001) proposed that similar brain areas were recruited during movement execution and motor imagery, these observations were based on qualitative comparisons based on a relatively limited sample of studies available at the time of writing. The present work, therefore, presents quantitative comparisons of the networks recruited in relation to motor imagery, providing a synthesis of results based on several hundred studies. This approach allowed us to examine the extent of the similarities and differences between these networks.

## 2 Methods

### 2.1 Datasets

Relevant neuroimaging papers were identified through two existing meta-analyses. Regarding movement execution and motor imagery, the data came from Hardwick et al. (2018) and for working memory all the papers included in this analysis were those included in Rodríguez-Nieto et al. (2022)’s work. A total of 697 papers were included (see Table 1).

**Table 1.** Data included in the meta-analyses.

Paradigm	Papers	Experiments	Participants	Foci
Movement execution	71	107	1687	1842
Motor imagery	134	216	3349	3214
Working memory	492	492	11,323	10,086
All	697	815	16,359	15,142

### 2.2 Data extraction and classification

Each paper was assigned a unique identifier based on the first author’s surname and the year of publication, with letters (a, b, c, etc.) appended to distinguish duplicates. Extracted data included the number of participants in each experiment and the coordinates of reported activations in MNI or Talairach space. Talairach coordinates were converted to MNI space using the Lancaster transform (Lancaster et al., 2007). Each paradigm was categorized as involving movement execution, motor imagery, or working memory. Additional details for each of the included papers can be found in the corresponding meta-analyses (Hardwick et al., 2018; Rodríguez-Nieto et al., 2022).

### 2.3 Data analyses

#### 2.3.1 General procedures

First, we conducted separate activation likelihood estimation (ALE) meta-analyses to identify the individual networks involved in experimental paradigms examining motor imagery, movement execution, and working memory. Next, we used conjunction analyses to examine the convergence and divergence between these networks. Conjunction analyses were calculated using the minimum statistic (Nichols et al., 2005); by overlaying the networks in a pairwise fashion, we identified the volume specific to each individual paradigm, and the volume activated across multiple paradigms. Regions consistently engaged across different paradigms were identified through pairwise conjunction analyses, and a final combined conjunction analysis highlighted the regions consistently recruited across all three paradigms. These results are detailed in Section 2 in the Supplementary Materials (Tables S4-S7, Figs. S1-S2). For contrast analyses, see Sections 3 and 4 in Supplementary Materials (Tables S8-S15, Fig. S3).

#### 2.3.2 ALE analyses

The analyses utilized the revised ALE algorithm (Eickhoff et al., 2009; Turkeltaub et al., 2002, 2012), which assesses whether the convergence of activation coordinates (foci) across experiments exceeds what would be expected by chance. Reported foci are represented as centres of 3D Gaussian probability distributions (Turkeltaub et al., 2002). In the revised algorithm, the width of these Gaussians is determined through empirical comparisons, modelling the increased spatial reliability associated with larger sample sizes by employing smaller Gaussian distributions (Eickhoff et al., 2009).

Foci from each experiment were aggregated across voxels to generate a modelled activation map (Turkeltaub et al., 2012). The combination of these modelled activation maps across experiments produced ALE scores, which indicate the convergence of coordinates for each location. These ALE scores were compared with a non-linear histogram integration based on the frequency of distinct modelled activation maps (Eickhoff et al., 2012), identifying areas where convergence exceeded chance expectations. ALE values were calculated exclusively for voxels with a probability of ≥10% of containing grey matter (Evans et al., 1994), as functional activations are primarily observed in grey matter regions. The results were thresholded at p < 0.05 (cluster-level family-wise error, corrected for multiple comparisons, with a cluster-forming threshold at voxel level p < 0.001) and reported at a voxel resolution of 2 x 2 x 2mm. Results are reported with a minimum cluster volume of 100 mm³ (i.e., ≥13 voxels, Beissner et al., 2013; Erickson et al., 2014; Turkeltaub et al., 2012).

#### 2.3.3 Conjunction analyses

Conjunction analyses synthesized the overlap between networks, under the conjunction null hypothesis (i.e., that at least one of the effects is absent). They were computed using the minimum statistic method (Nichols et al., 2005), which compares the voxel-wise minimum value across volumes, and returns as output the lower value for each voxel. This ensures that only voxels showing significant effects in all contrasts are retained, thereby identifying regions consistently engaged across conditions. A minimum cluster volume of 100 mm³ was applied (Beissner et al., 2013; Erickson et al., 2014; Turkeltaub et al., 2012).

#### 2.3.4 Volume matched analyses

Recognizing that differences in the number of overall voxels in each network could impact the size of identified overlap between them, “volume-matched analyses” (Hardwick et al., 2015) were conducted. Here, two networks were compared, with the smaller network’s volume designated as the target size. The larger network’s volume was iteratively reduced in size by increasing its threshold until the volume difference was minimized. This threshold was then applied to the larger network, resulting in a volume-corrected version closely matching the smaller network’s voxel count. This process identified regions consistently implicated in a paradigm, while also controlling differences in resulting volumes between different analyses.

#### 2.3.5 Mask analysis by brain area

This analysis, inspired by Jeannerod’s (2001) original qualitative assessment of the recruitment of different brain regions by motor imagery and movement execution, examined the overlap for each task paradigm within different areas of the brain. We first identified regions that were implicated in the main meta-analysis of motor imagery (i.e. Area ∩ motor imagery), then examined the extent to which movement execution and working memory overlapped in these regions. Further analysis examined whether one of these paradigms showed greater recruitment of one of these specific sub-regions than the other paradigm. Volume-matched analysis was also conducted for this analysis (see Table 3 for the results).

Regions to be examined were identified based on the brain areas for which we observed maximum activation peaks during the main analysis of the neural network of motor imagery. Templates of the areas to be studied were identified from three different sources. Brodmann area templates were taken from MRIcroGL (Rorden, 2025). Cortical motor areas were examined using the Human Motor Area Template (HMAT; Mayka et al., 2006). Templates of cerebellar regions were extracted from the Spatially Unbiased Infratemporal Template (SUIT; Diedrichsen et al., 2009). Given that the DLPFC has been considered to play an important role in motor imagery across different models (Glover & Baran, 2017; Jeannerod, 2001), we created a template for this region based on the designation by Petrides (2005), by combining the available templates for Brodmann Areas 9 and 46. Similarly, we note that although area 2 is part of S1, the two zones have been considered in their entirety for the purposes of this analysis, as there is no total overlap. Cross-referencing these templates with the regions implicated in the main meta-analysis of motor imagery identified 16 areas for analysis in further depth (see Table 2).

**Table 2.** Brain atlases used in the analyses, and their origins.

Area	Atlas	Source
*DLPFC*	Brodmann atlas (Merge of Areas 9 and 46)	MRIcroGL
*Area 2*	Brodmann atlas	MRIcroGL
*Area 7*	Brodmann atlas	MRIcroGL
*Area 32*	Brodmann atlas	MRIcroGL
*Area 40*	Brodmann atlas	MRIcroGL
*Area 44*	Brodmann atlas	MRIcroGL
*Area 45*	Brodmann atlas	MRIcroGL
*Area 48*	Brodmann atlas	MRIcroGL
*PMd*	HMAT	LRNLAB.org website
*PMv*	HMAT	LRNLAB.org website
*S1*	HMAT	LRNLAB.org website
*Cereb_VI*	SUIT atlas	SPM Anatomy Toolbox
*IPL_PF*	Cytoarchitectonic map of the human IPC	SPM Anatomy Toolbox
*IPL_PFcm*	Cytoarchitectonic map of the human IPC	SPM Anatomy Toolbox
*IPL_PFt*	Cytoarchitectonic map of the human IPC	SPM Anatomy Toolbox
*AIPS_IP1*	Cytoarchitectonic map of the human IPS	SPM Anatomy Toolbox

Conjunctions were made between the template provided for each area of the brain and motor imagery (e.g. performing the conjunction DLPFC ∩ motor imagery revealed the regions of the DLPFC specifically recruited during motor imagery). Further conjunctions were then conducted to identify the overlap in a given brain region between motor imagery and movement execution/working memory (e.g. the conjunction “DLPFC ∩ motor imagery ∩ working memory” would reveal the regions of the DLPFC that are recruited by motor imagery that are also recruited during working memory tasks). The number of voxels identified in each analysis was converged to percentages, calculated relative to the volume of a given brain area that was recruited during working memory (allowing interpretations such as “of the volume of the DLPFC” that is recruited by motor imagery, 92% is also recruited by working memory). These steps then allowed the calculation of the “observed difference” in the recruitment of each area by each paradigm (i.e. calculated as the difference in the percentage of recruitment of the DLPFC identified in the conjunctions “DLPFC ∩ motor imagery ∩ movement execution” and “DLPFC ∩ motor imagery ∩ working memory”).

#### 2.3.6 Labelling

The results were anatomically labelled based on their most likely macro-anatomical and cytoarchitectonic/tractographically assessed locations using the SPM Anatomy Toolbox 2 extension (Eickhoff et al., 2005, 2006, 2007). Additionally, functional labels for motor regions were assigned using the human motor area template (HMAT) defined by Mayka et al. (2006). Brodmann area labels were added using the MRIcron Anatomical Template. The reported coordinates were based on peak maxima in MNI space.

## 3 Results

### 3.1 Paradigm-specific analyses

#### 3.1.1 Movement execution

**Fig. 1.**
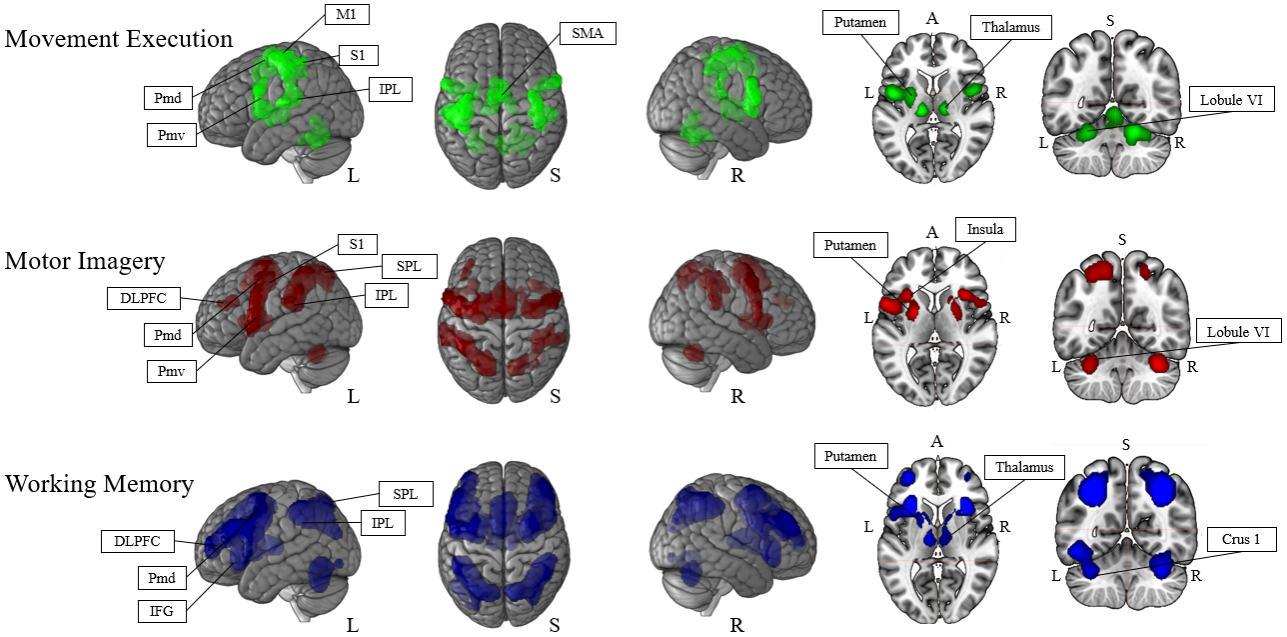
Quantitative meta-analyses of the three paradigms.

The analysis of movement execution included 71 experiments reporting 1842 foci recorded from a total of 1687 participants. Movement execution identified the network with the smallest overall volume of the meta-analyses conducted, with a total of 11,229 voxels (a comparison of the volumes across networks is presented in Fig. 1; see also Supplementary Materials Section 1, Tables S1-S3 for detailed summaries). Bilateral recruitment in the sensorimotor and premotor areas was identified and included the left supplementary motor area (SMA), bilateral ventral premotor cortex (PMv), and left lateralized dorsal premotor cortex (PMd). Two bilateral clusters around the primary motor and somatosensory areas were found. Subcortically bilateral areas in the thalamus, cerebellum (including lobule VI), and putamen were also recruited.

#### 3.1.2 Motor imagery

Motor imagery included 134 experiments reporting 3214 foci recorded from a total of 3349 participants. Motor imagery recruited a total of 16,194 voxels, primarily recruiting bilateral premotor areas as well as left lateralized recruitment of the DLPFC. The largest cluster encompassed the left ventral and dorsal premotor cortex and extended to the insula lobe. In the right hemisphere, another cluster included ventral and dorsal premotor cortex, the insula lobe, and the putamen. Recruitment of the parietal lobule was identified in both hemispheres. Further bilateral clusters were identified in the cerebellum (lobule VI).

#### 3.1.3 Working memory

The analysis of working memory involved 492 experiments reporting 3214 foci recorded from a total of 11,323 participants with a total volume of 27,724 voxels The analysis revealed a fronto-parietal network. The largest cluster was detected in the left hemisphere, spanning frontal (inferior and middle frontal gyri), and subcortical (putamen, thalamus) regions. A similar cluster was detected in the right hemisphere. Parietal clusters were identified in both hemispheres, extending across the superior and inferior parietal lobules. Cerebellar clusters were found bilaterally around the Crus I area.

### 3.2 Conjunction analyses

**Fig. 2.**
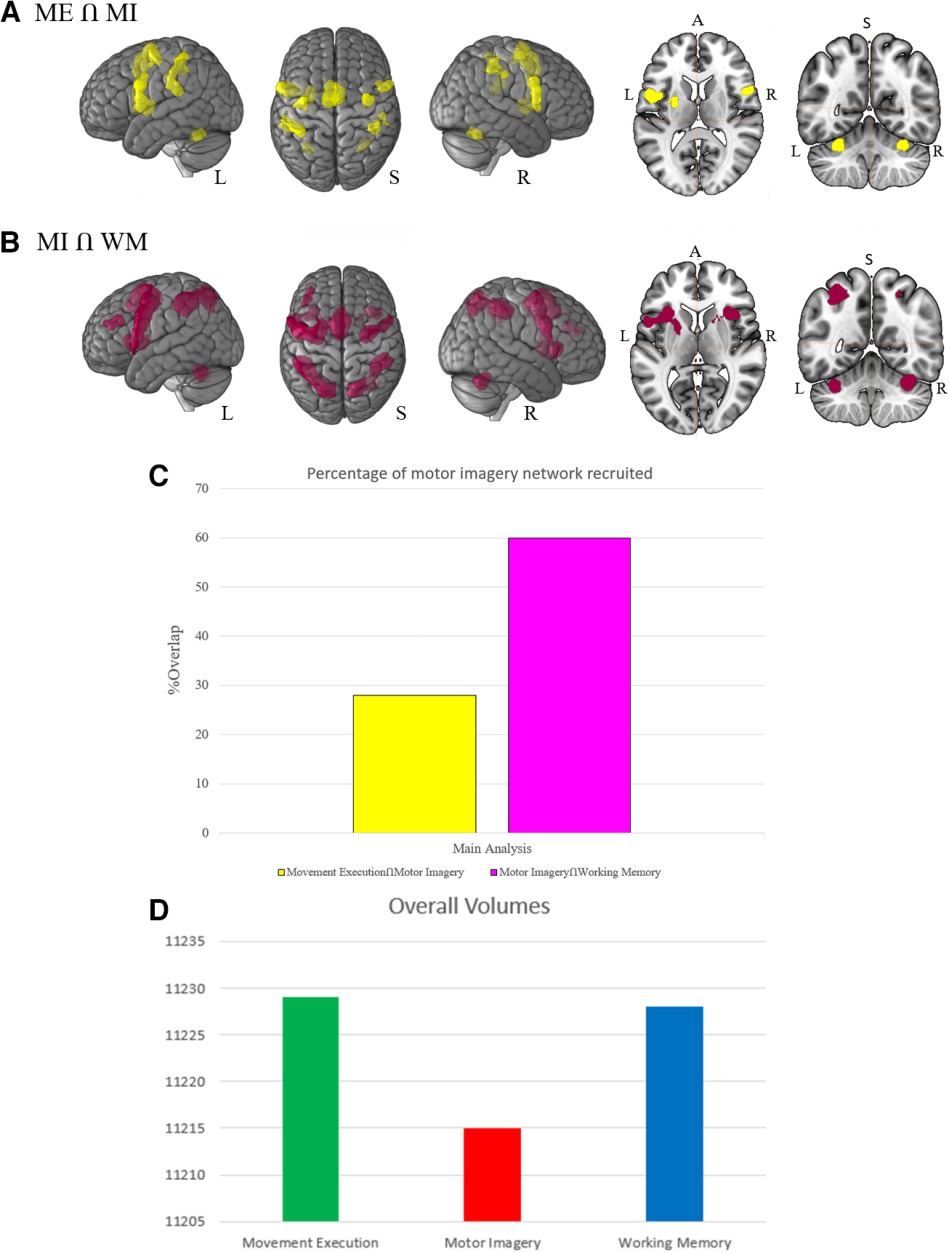
Conjunction analyses conducted across combinations of the paradigms. (A) Represents the network activated during motor imagery and movement execution (yellow). (B) Presents the network activated during motor imagery and working memory (purple). (C) Comparison of the overlap between the motor imagery network and the other paradigms. While 60% of the motor imagery network was also recruited by working memory, compared with only 28% of the movement execution network, we note that considerable differences in the overall sizes of each network (D) may have contributed to this effect.

Minimum-statistic conjunction analyses were performed to identify regions that were consistently activated across the different paradigms (Fig. 2; see also Supplementary Tables S4–S7).

#### 3.2.1 Movement execution ∩ motor imagery

A conjunction analysis of motor imagery and movement execution revealed a network consisting of bilateral sensorimotor and premotor cortical clusters, along with smaller subcortical clusters in the putamen and cerebellum (Fig. 2). In the premotor regions, a medial, superior cluster encompassed the bilateral preSMA and SMA proper. Additional bilateral clusters spanned the ventral premotor cortex and extended to the left dorsal premotor cortex. Posterior to these premotor clusters, two bilateral clusters were identified, each involving the primary somatosensory cortex. Subcortically, the left putamen and bilateral cerebellum (lobule VI) were consistently activated across both analyses.

#### 3.2.2 Working memory ∩ motor imagery

Consistent activations across motor imagery and working memory were identified mainly in the premotor regions (Fig. 2). One cluster included the left ventral and dorsal premotor cortex and extended to the putamen, while another included the right ventral and dorsal premotor cortex. A small cluster included the right superior parietal lobule and the right precuneus. Another cluster common to both paradigms involved the left DLPFC. Subcortically, conjunction revealed common activation of the right insula lobe, the right putamen but also bilateral activations in the cerebellum.

#### 3.2.3 Comparison of overlap with the motor imagery network

Results from the conjunction analyses presented above were considered in relation to the extent to which each network overlapped with the network involved in motor imagery. These results indicated that the network for motor imagery had greater overlap with working memory (60% of voxels in the motor imagery network) than with movement execution (28% of voxels in the motor imagery network; difference of 42%).

### 3.3 Volume-matched control analyses

A “volume-matched” analysis was conducted to control for possible differences in overlap between the two paradigms that was simply due to the fact that working memory recruited more voxels overall than movement execution. The meta-analysis for movement execution was identified as having the smallest overall volume (11,229 voxels). Iteratively increasing the threshold applied to the networks allowed us to reduce their total volumes to be as close to the volume of the movement execution as possible (Motor imagery: 11,215 voxels, Working memory: 11,228 voxels).

**Fig. 3.**
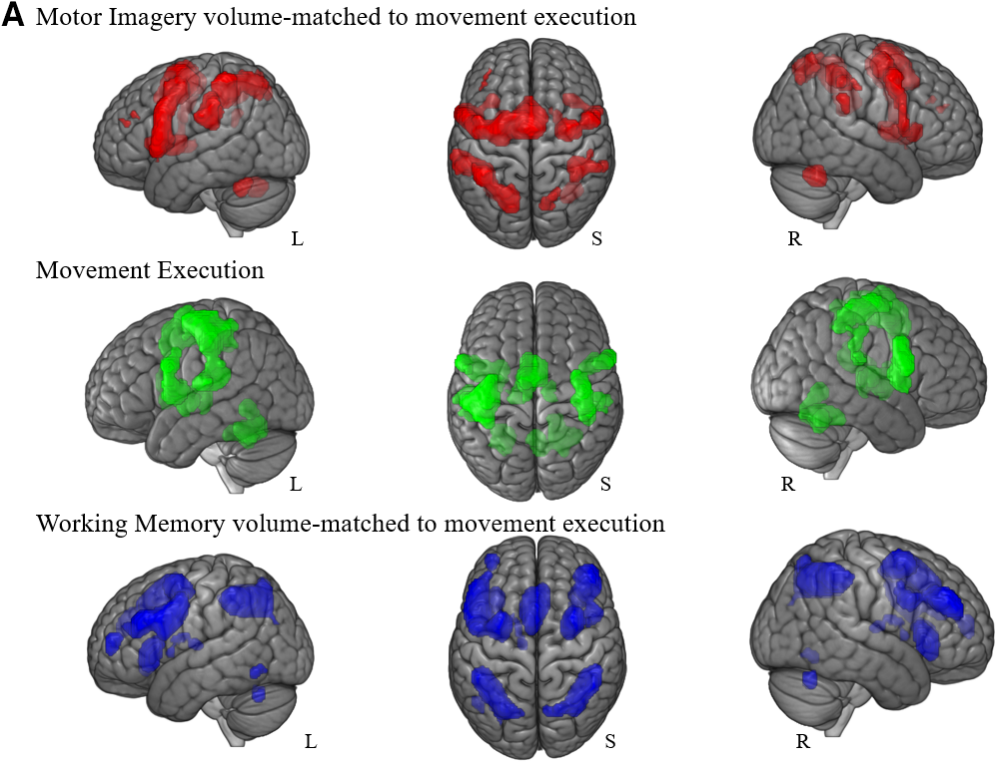

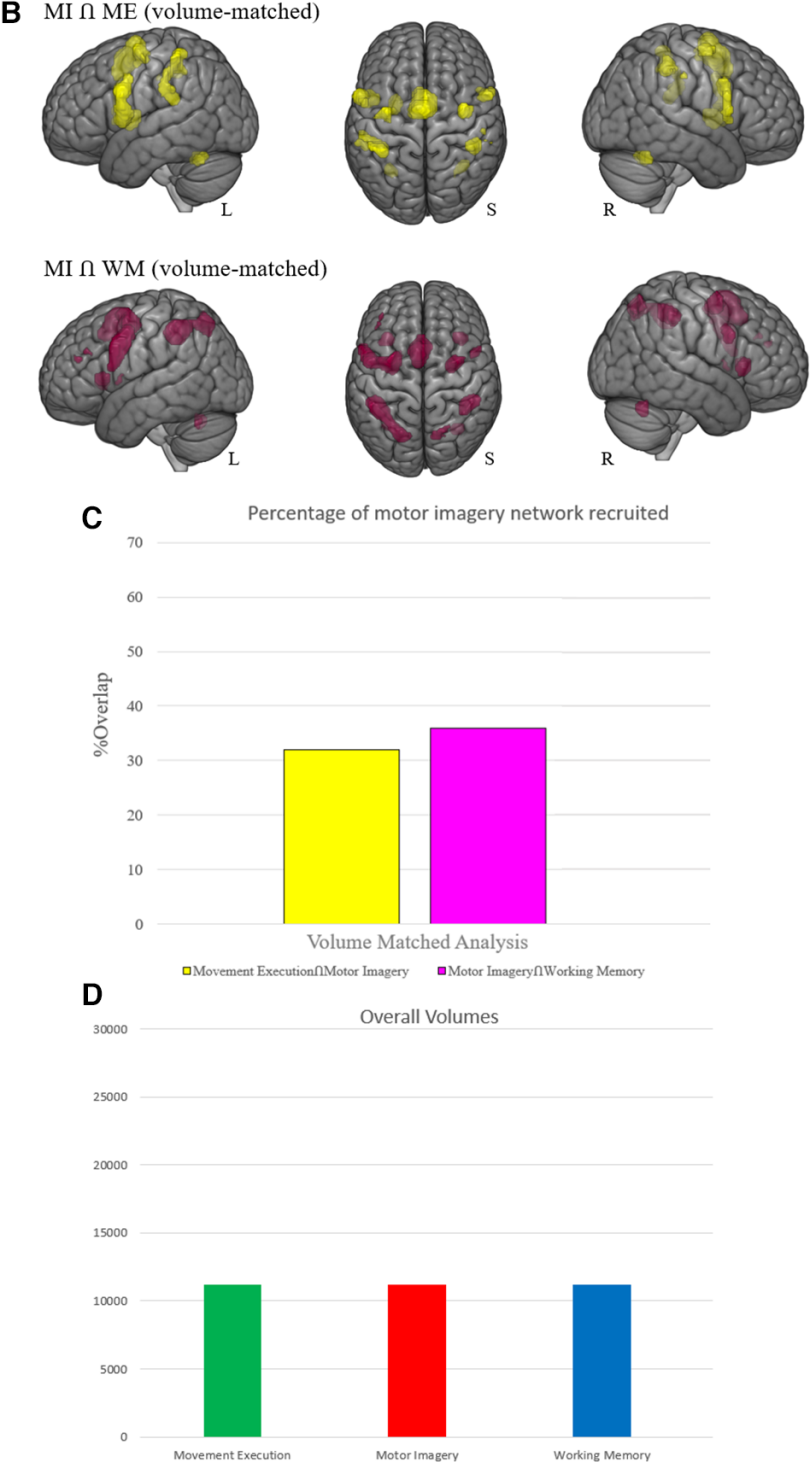
Volume-matched brain areas and overlap percentage. (A) represents the network for the volume-matched analysis activated during each paradigm and during the conjunction between motor imagery and movement execution in yellow and the conjunction between motor imagery and working memory in purple. While the volume-matching approach (B) considerably reduces the difference in the extent of the overlap between the networks (c.f. Fig. 2C.), there is still greater overlap between motor imagery and working memory than with motor imagery and movement execution. (C) Represents the overall volume for each paradigm to match with movement execution, while (D) demonstrates that the volume-matching procedure led to minimal differences between the number of voxels in each network examined.

While we did not observe any major differences in terms of the regions involved in the conjunction analyses, this did affect the overall volumes as identified in the overlap analysis. In percentage terms, the volume-matched motor imagery network shared 32% of its total volume with movement execution, and 36% of its volume with working memory (Fig. 3). Thus, both our main and volume-matched analyses indicated that there was a greater overlap between motor imagery and working memory than for motor imagery and movement execution.

### 3.4 Mask analysis by brain area

**Fig. 4.**
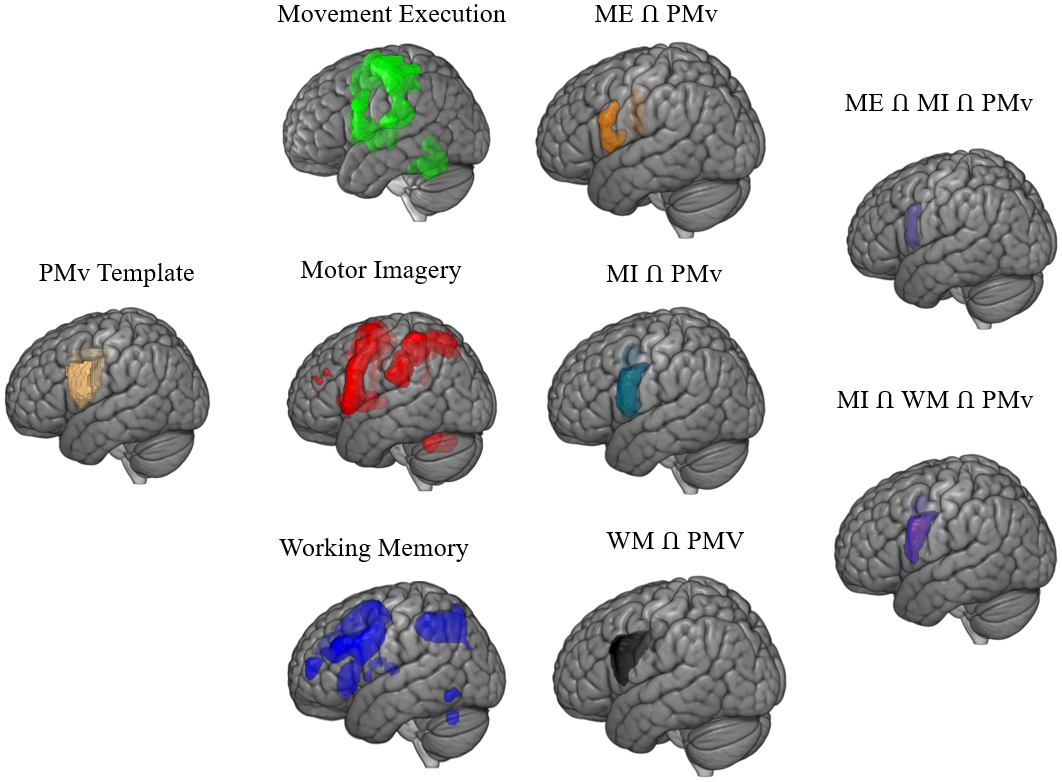
Example of mask analysis with ventral premotor cortex (PMv).

This analysis makes it possible to determine whether certain areas of the brain are more common to motor imagery and working memory, or whether they overlap more with movement execution (see Table 3 and Fig. 4).

**Table 3.** Volume of activation (in voxels) in common with motor imagery by different areas of the brain.

		Full network analysis				Volume-matched analysis			
Brain area	Total volume	Volume area∩MI	Volume area∩MI∩WM	Volume area∩MI∩EX	Diff overlap	Volume area∩MI	Volume area∩MI∩WM	Volume area∩MI∩EX	Diff overlap
AIPS_IP1	2805	649	640 (98.6%)	37 (5.7%)	603 (+92.9%)	482	428	34	**394**
Area 7	6194	1394	1 269	0	**1269**	829	440	0	**440**
Area 32	4009	327	313	70	**243**	248	225	69	**156**
Area 45	3566	89	85	0	**85**	20	11	0	**11**
DLPFC	8069	141	130	0	**130**	34	17	0	**17**
Area 40	3975	1789	1 305	279	**1026**	1376	757	261	**469**
PMd	6599	2794	1 845	240	**1605**	2189	853	220	**633**
Area 44	2320	506	463	195	**268**	331	236	164	**72**
IPL_PFcm	2555	753	16	122	-106	488	0	99	-99
Cereb_VI	9020	734	492	305	**187**	493	133	210	-77
S1	4288	645	235	437	-202	296	28	216	-188
Area 2	1824	763	327	419	-92	487	98	308	-210
IPL_PFt	3497	1580	735	553	**182**	1068	345	390	-45
IPL_PF	5069	963	205	67	**138**	569	60	49	**11**
Area 48	19 932	2177	789	725	**64**	1343	254	511	-257
PMv	5838	2038	1 281	1 024	**257**	1506	544	834	-290

For “difference overlap” values, positive values (in bold) indicate greater overlap between motor imagery and working memory, while negative values indicate greater overlap between motor imagery and movement execution. Percentages are in relation to the volume of a given area that are shared between motor imagery and another paradigm (e.g. for the AIPS_IP1, 98.6% of the region recruited by motor imagery is also recruited by working memory, while only 5.7% of the area recruited during motor imagery is also recruited during movement execution).

Results of this analysis indicate that the majority of brain areas recruited during motor imagery were activated to a greater extent by working memory than by movement execution. In particular, several associative regions, including frontal (DLPFC, Area 45) and parietal (Area 7) areas, were recruited uniquely by motor imagery and working memory tasks. By contrast, relatively few regions of the motor imagery network (S1/Area 2 and the IPL_PFcm) demonstrated a greater recruitment by movement execution than by working memory tasks.

The volume-matched analysis for this section revealed a similar overall pattern of results with the full volume analysis. With this control analysis, we see more overlap between motor imagery and movement execution in sensorimotor regions (S1, Area 2, PMv, Cerebellum) and more overlap between motor imagery and working memory in associative regions (frontal areas).

## 4 Discussion

The similarity between the brain networks involved in movement execution and motor imagery has already been the topic of numerous studies (Decety et al., 1994; Grèzes & Decety, 2001; Jeannerod, 2001; Lotze & Halsband, 2006). In particular, Jeannerod (2001) proposed “Motor Simulation Theory” based on a qualitative assessment of the similarity between the brain regions recruited during movement execution and motor imagery. Later quantitative syntheses of the literature have mapped their respective brain networks, identifying both notable similarities and differences between movement execution and motor imagery (Hardwick et al., 2018; Hétu et al., 2013). More recently emerging theories have proposed a plausible explanation for these differences, arguing that motor imagery may place a greater emphasis on cognitive resources that have previously been considered. Specifically, the Motor-Cognitive Model proposes that motor imagery may use central executive resources in a fashion similar to working memory (Glover et al., 2020; Glover & Baran, 2017). The present study, therefore, conducted a series of analyses to quantify the similarity in the volumes activated during movement execution, motor imagery, and working memory. Our results indicate that there is greater overlap between the brain regions involved in working memory and motor imagery than movement execution. While our main analysis shows stronger recruitment of working memory-related network, the volume-matched analysis reveals that the overall difference is relatively modest, with a slight advantage for working memory over movement execution. Taken together, these findings suggest that motor imagery may place a greater reliance on central executive resources than has previously been considered.

These results can be integrated with the recent neurophysiological evidence provided by Dekleva et al. (2024). While our meta-analysis highlights that motor imagery overlaps more strongly with working memory than execution, Dekleva et al. (2024) demonstrate that motor cortex nevertheless retains the dynamical structure of executed movements during imagery, although reoriented into an output-null subspace. Together, these findings suggest that motor imagery sits at the interface of motor and cognitive systems: it preserves core motor dynamics, but sustaining these covert representations requires additional recruitment of executive resources, consistent with the Motor-Cognitive model.

### 4.1 Key observations

Jeannerod’s seminal paper describing the Motor Simulation Theory (2001) involved qualitative comparisons identifying similarities between the brain networks identified during movement execution and motor imagery. In the present manuscript, a series of conjunction analyses revealed that motor imagery shares a significantly larger proportion of its neural network with the network recruited during working memory than with the network recruited during movement execution. Volume-matched analyses confirmed that this result is not simply due to differences in the overall sizes of the volumes for each network. Further investigation identified a series of frontal and parietal regions that showed a greater volume of recruitment during working memory than during movement execution. This is in keeping with the view that motor imagery and working memory share underlying mechanisms involving temporary storage, manipulation, and updating of information based on context, similar to working memory tasks (A. Baddeley, 2012; A. D. Baddeley & Hitch, 1994).

Our findings align with the Motor-Cognitive model (Glover et al., 2020; Glover & Baran, 2017), which proposes that motor imagery engages executive resources similar to those involved in working memory. Supporting this idea, studies have shown that motor imagery is more affected by interfering tasks engaging executive functions than movement execution, highlighting that motor imagery relies on cognitive processes (Glover & Baran, 2017). Additionally, research using transcranial magnetic stimulation has demonstrated that disrupting regions involved in executive functions specifically slows down motor imagery without affecting movement execution (Martel & Glover, 2023), further emphasizing that motor imagery is not only a sensory-motor simulation, but also depends on broader cognitive mechanisms related to information processing and management.

### 4.2 Network-level conjunction analyses

Conjunctions were performed to identify those regions that are common to both motor imagery and movement execution, as well as those that are common to both motor imagery and working memory. By observing these differences and similarities, it would seem that pre-SMA and SMA are specific to the “movement” part of motor imagery. Indeed, in his meta-analysis, Hétu et al. (2013) linked the SMA to the processing of complex information associated with movement initiation, and visuo-spatial transformations. However, the specific role of the SMA during motor imagery could vary depending on the type of motor imagery being performed.

In contrast, the ventral and dorsal premotor cortices play a role in the planning, preparation, and execution of movement (Hoshi & Tanji, 2007). Movement planning has various components (Sensory integration, Goal selection, Motor planning, Preparation for execution). During this planning, it is essential to gather information about the external environment, store it in memory, and then use it wisely to prepare and then execute the movement. Working memory works in a very similar way. Indeed, working memory tasks require individuals to maintain task-relevant information in the short-term memory, ensuring it is available for later recall or recognition. These tasks also involve revising this information based on the task’s context, often requiring manipulation of the data as part of the process (A. Baddeley, 2012; A. D. Baddeley & Hitch, 1994).

**Fig. 5.**
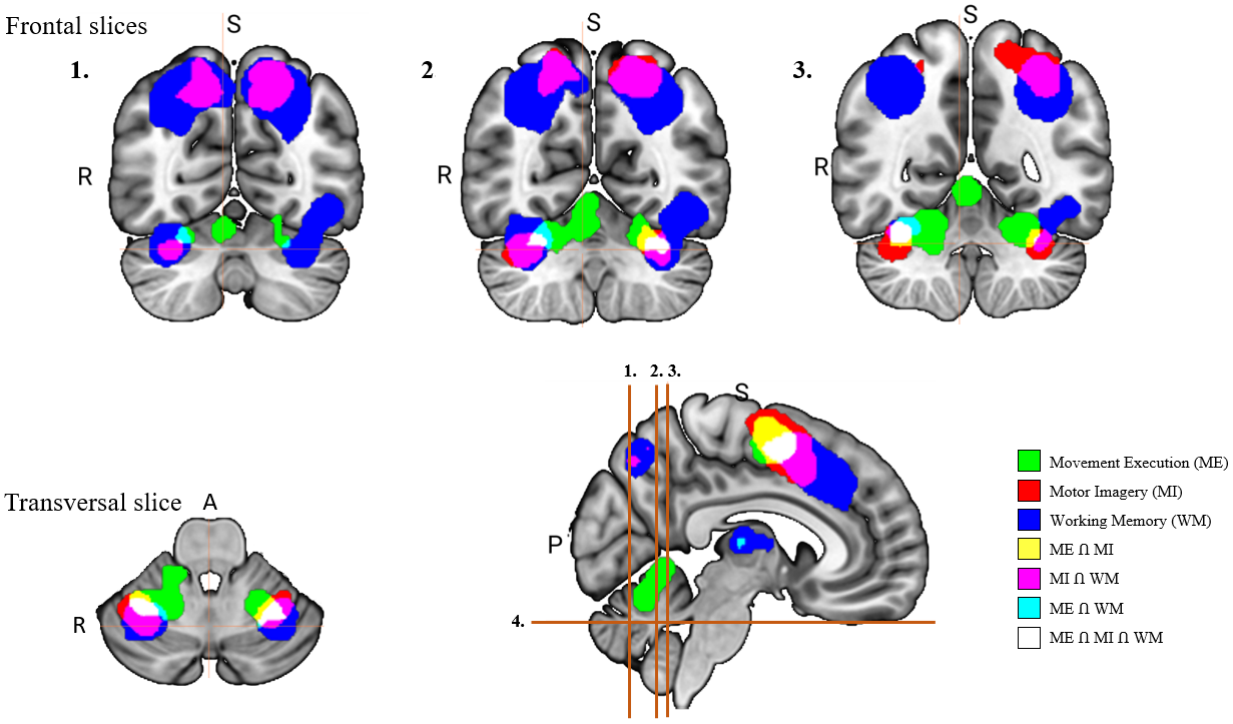
Representation of the cerebellar activity for the three paradigms.

Analyses then revealed that all three paradigms activated cerebellar lobule VI, whereas only motor imagery and working memory recruited Lobule VIIa (Fig. 5). However, we note that just because several paradigms activate the same area of the brain, it does not necessarily mean that they do so for the same function. Hardwick et al. (2018) also highlighted in their meta-analysis a shared activation of lobule VI during both movement execution and motor imagery; however, motor imagery lacked the somatotopic activation identified during movement execution, suggesting that these overlapping activations may serve differing functions. Regarding its activation during working memory, recent studies have also shown that the cerebellum plays a role in higher cognitive functions (McDougle et al., 2022). Research in people with “cerebellar cognitive affective syndrome”, caused by damage or dysfunction of the cerebellum, has linked this region to deficits in executive function, particularly working memory (Beuriat et al., 2020; Hayter et al., 2007; Ramnani, 2006). Notably, individuals with lesions in lobule VI exhibit impairments in working memory, while other executive functions remain unaffected (Beuriat et al., 2020). This aligns with Rodríguez-Nieto et al.’s (2022) meta-analytic study that has consistently associated cerebellar activation with tasks involving working memory, but not with other aspects of executive function. Importantly, recent work has emphasized that activations in Crus I are more consistently associated with working memory, in line with its connectivity to the fronto-parietal control network (Buckner et al., 2011; Marek et al., 2018), whereas lobule VI is often engaged across both motor and cognitive domains and may participate in the integrative somato-cognitive action network (Gordon et al., 2023). This further reinforces the connection between motor imagery and working memory. The distinct motor and cognitive roles of the cerebellum may correspond to different models of motor imagery: the “motor” interpretation of cerebellar activity during motor imagery aligns more closely with Motor Simulation Theory, while its potential involvement in working memory supports the Motor-Cognitive model.

Finally, DLPFC was only activated during motor imagery and working memory. It is also at the centre of discussions about the Motor-Cognitive model (Martel & Glover, 2023). According to this model, the DLPFC may provide central executive resources for motor imagery, functioning in a manner similar to its role in working memory. The Motor Simulation Theory also acknowledges a possible involvement of the DLPFC, but the possible function of this region is only briefly described, with the suggestions that the DLPFC may be involved in movement preparation and short-term information storage. In contrast, the Motor-Cognitive model offers a more explicit and developed role for the DLPFC within motor imagery. The concepts proposed by both models align with recent research emphasizing the existence of “motor working memory” (Bardakan et al., 2022; Hillman et al., 2024; McDougle & Hillman, 2025). Indeed, these findings reflect distinctions in representational formats observed in other domains, suggesting that motor working memory shares a fundamental computational structure with other working memory subsystems.

### 4.3 Analyses by brain region

Previous analyses have shown that there was indeed a difference in overlap between the two conditions (full network analysis and volume-matched analysis). However, these analyses did not determine in which regions of the brain these differences are found. Thanks to the analysis by brain region, we were able to observe whether specific sub-regions of the motor imagery network shared greater co-activation with regions involved in movement execution or working memory. Notably, in both our main and volume-matched analyses, the majority of sub-regions within the motor imagery network had greater overlap with regions involved in working memory than with movement execution; here we limit our discussion to those regions that were consistent across these two analyses.

#### 4.3.1 Regions of the motor imagery network with greater overlap with movement execution than working memory

The regions showing greater overlap between motor imagery and movement execution primarily encompassed a sensorimotor network, including the primary somatosensory cortex (S1, Brodmann area 2 (BA2)) and the dorsal (PMd) and ventral premotor cortices (PMv).

Within this network, S1/BA2 could contribute to the reactivation of sensory consequences associated with movement, such as tactile and proprioceptive feedback (Naito et al., 2002; Porro et al., 1996). Even in the absence of actual execution, these somatosensory areas recreate the sensory state that would normally accompany the movement, providing a foundation for the vividness and realism of motor imagery (Jeannerod, 2001; Kilteni et al., 2018). The engagement of these regions supports the view that motor imagery relies not only on simulating motor commands but also on reinstating their sensory outcomes, maintaining coherence between the imagined and executed states of action.

In close interaction with these sensory cortices, the premotor areas (PMd and PMv) are central to generating and structuring internal motor representations. The dorsal premotor cortex (PMd) is known for its role in planning and selecting movements based on external sensory cues (Cisek & Kalaska, 2010; Hoshi & Tanji, 2007). During motor imagery, its recruitment indicates that the brain engages similar preparatory mechanisms as in actual motor planning (Gerardin et al., 2000; Hétu et al., 2013). The ventral premotor cortex (PMv), by contrast, contributes to the understanding and representation of goal-directed actions. Homologous to the primate area F5, which contains mirror neurons, PMv integrates sensory information and motor commands to construct coherent representations of actions, whether observed, executed, or imagined (Fogassi et al., 2005; Rizzolatti & Craighero, 2004).

The functional interplay between these regions, with S1 reactivating sensory feedback, PMd generating motor plans, and PMv representing action goals, suggests that motor imagery emerges from sensorimotor integration across cortical levels.

#### 4.3.2 Regions of the motor imagery network with greater overlap with working memory than movement execution

Beyond the core sensorimotor system, motor imagery also recruits a fronto-parietal associative network encompassing the dorsolateral prefrontal cortex (DLPFC), anterior cingulate cortex (Brodmann area 32 (BA32)), inferior frontal gyrus (Brodmann area 45 (BA45)), superior parietal lobule (Brodmann area 7 (BA7)), and supramarginal gyrus (Brodmann area 40 (BA40)). These regions overlap more strongly with the working memory network than with movement execution, indicating that motor imagery engages high-level cognitive and executive mechanisms necessary to maintain and manipulate internal representations of action.

The DLPFC has previously been the subject of research linking working memory and motor imagery (Martel & Glover, 2023). It is involved in frontal-executive functions related to action preparation (Mars & Grol, 2007), which are thought to share neural substrates with motor imagery. The recruitment of the DLPFC during motor imagery may be attributed to working memory demands, which are considered distinct from the frontal-executive functions of the DLPFC that are engaged during more complex movement tasks (Rottschy et al., 2012). An alternative account is that the DLPFC supports movement inhibition, given its established role in suppressing overt actions (Blasi et al., 2006; Coxon et al., 2016). However, this interpretation contradicts the specific predictions of simulation theory, which attributes inhibition during motor imagery specifically to orbitofrontal and cingulate prefrontal regions, but not to the DLPFC (Jeannerod, 2001). From this perspective, motor imagery could rely on the simulation model, but include an inhibitory mechanism arising from the DLPFC to prevent execution. While inhibition is undoubtedly necessary when movements are internally simulated but not carried out, it is unlikely to fully account for the observed overlap between motor imagery and working memory (Hampshire et al., 2010). Moreover, the anatomical boundaries of the DLPFC are not sharply defined and often overlap with adjacent regions such as the inferior frontal gyrus, which has also been implicated in inhibitory control (Hampshire et al., 2010). From this perspective, the involvement of lateral prefrontal areas may better reflect broader executive demands such as the active maintenance and manipulation of motor representations (D’Esposito et al., 1995), rather than inhibition alone. Thus, the overlap with the working memory network may be more parsimoniously interpreted in terms of shared executive resources, rather than as a mere byproduct of suppressing overt movement.

The anterior cingulate cortex (BA32) complements the DLPFC by linking cognitive and motor domains. This region is anatomically positioned to integrate signals from both the hippocampus and premotor areas (Rolls, 2019), enabling it to coordinate action monitoring, error detection, and the retrieval of relevant episodic or semantic information during imagery. Its dual involvement in memory and motor control processes underscores the hybrid nature of motor imagery, combining executive and embodied components (Strotzer, 2009).

In the inferior frontal gyrus (BA45), a region traditionally associated with Broca’s area, activity during motor imagery supports the integration of semantic and sensorimotor representations. BA45 acts as a high-level interface that connects cognitive understanding with motor intention (Binkofski & Buccino, 2004), particularly for goal-oriented or symbolic actions. Its role in non-spatial working memory (D’Esposito et al., 1998) and mirror neuron activation (Guillot et al., 2014; Rizzolatti et al., 1998) suggests that it contributes to retrieving and evaluating the meaning of internal representations within a given context.

The parietal components of this network (BA7 and BA40) are essential for linking perception and action through spatial and sensorimotor integration. BA7, corresponding to the superior parietal lobule and intraparietal sulcus, supports visuo-perceptual coordination and the transformation of sensory input into motor coordinates (Strotzer, 2009). Meanwhile, BA40 (the supramarginal gyrus) integrates sensory and motor information at the parietal–temporal junction, supporting action planning, imitation, and abstract reasoning (Mara, 2017; Oztop et al., 2013). Both regions host mirror neurons that facilitate the anticipation and simulation of observed or imagined actions, and their coactivation with frontal regions reinforces the dynamic coupling between perceptual prediction and executive monitoring.

Taken together, the DLPFC, anterior cingulate cortex, inferior frontal gyrus, and parietal cortices form a distributed fronto-parietal control network. This network supports the generation, maintenance, and manipulation of internal motor representations while preventing overt movement. It ensures that motor imagery remains coherent, goal oriented, and contextually appropriate, reflecting a continuous interaction between cognitive control and embodied simulation.

### 4.4 Strengths and limitations

Although our meta-analysis provides new insights into the relationship between motor imagery, movement execution, and working memory, some limitations must be acknowledged. First, the data included are from pre-existing meta-analyses (Hardwick et al., 2018; Rodríguez-Nieto et al., 2022), and as the collection of articles stopped in June 2017 and December 2019 for these papers, respectively, articles from more recent literature were not included. We note, however, that we have no reason to expect they would significantly alter the results presented here. Similarly, as our analyses were based on meta-analytic approaches, our results rely on the availability and quality of the primary studies compiled in these works. Therefore, as is the case for all meta-analyses, publication bias and methodological differences between the original MRI/PET studies contributing to these works may influence the results.

Secondly, although the use of ALE (Activation Likelihood Estimation) makes it possible to identify common activations between the paradigms studied, this method does not reflect individual variations or the temporal dynamics of neuronal activity. In addition, activation coordinates are often reported at group level, which may mask significant inter-individual differences. This is particularly notable given that there is estimated to be considerable variability in the ability that individuals have to perform imagery (Wright et al., 2024), which may affect the associated brain networks activated. However, meta-analytical approaches such as ALE provide a pragmatic solution to the limitations of individual neuroimaging studies, which are typically underpowered (Szucs & Ioannidis, 2020). Moreover, as ALE focuses on peak coordinates, which represent the strongest activations in individual studies, this provides confidence that our results are representative of the “core” networks involved in each associated behaviour. However, a limitation of the volume-matched analysis is that while it controls for network-size bias, it does so by applying un-equal thresholds across networks. We followed this approach because it provides a pragmatic approach to allow volume-based comparisons across networks of different sizes, as in previous ALE studies (Hardwick et al., 2015, 2018). Nonetheless, by using a more conservative threshold for motor imagery and working memory, the analyses should still be highly representative of regions that are unambiguously associated with the respective task paradigms. Furthermore, the classification of paradigms into three distinct categories (motor imagery, movement execution, and working memory) simplifies a more nuanced reality. There are variations within each category, particularly between the different types of motor imagery and working memory tasks, which have not been fully explored in this analysis. While analyses of relevant sub-categories would have been of interest, unfortunately, the availability of relevant information in the primary literature places limitations on this. For example, previous research showed that 60% of motor imagery studies did not provide enough information to allow clear identification of the modalities and perspectives of imagery being used (Van Caenegem et al., 2022). Creating sub-categories in the present study would, therefore, have been limited, given that the majority of papers do not provide enough information to allow detailed inspection of these points. We hope that efforts to improve the quality of study reporting (e.g. Moreno-Verdú et al., 2024) will allow the inclusion of such detailed sub-analyses in future work.

Finally, our analyses do not allow us to establish causal relationships between the neural networks involved in these three processes. Further experimental studies, in particular using non-invasive brain stimulation or real-time neuroimaging protocols, would be necessary to investigate these relationships further.

## 5 Conclusion

Our results indicate that the brain network recruited during motor imagery shares a greater volume with working memory than with movement execution. These findings suggest that executive resources may play a greater role in motor imagery than has previously been appreciated. Activation of frontal and parietal regions involved in working memory during motor imagery suggests similarities between these two processes; future studies could further explore these links and their applications during experimental studies.

## Supplementary Material

Supplementary Material

## Data Availability

Data from all analyses are available at https://github.com/evancaenegem/ALE-WM-vs-MI.
