# Supplementary material for: Greater neural overlap between motor imagery and working memory than with movement execution: A meta-analytic comparison
Source: Imaging Neurosci (Camb). 2026 Jan 12;4:IMAG.a.1095. doi: 10.1162/IMAG.a.1095 (PMC12797143; doi:10.1162/IMAG.a.1095)
Supplement: Supplementary Material [file IMAG.a.1095_supp.pdf]

Supplementary Material

Section 1: Main analyses

Table S1: Meta-analysis of Movement Execution (n=71)

Cluster Voxels	Z-score	Macroanatomical Location	Cytoarchitectonic/ Tractographic Label	MNI Coordinates x	y	z
3077	8.13	L Postcentral Gyrus (S1)	L Area 3b	-38	-30	50
	6.60	L Rolandic Operculum (48)	L Area OP1 [SII]	-46	-26	18
	5.31	L SupraMarginal Gyrus (48)	L Area OP1 [SII]	-52	-26	28
	5.03	L Precentral Gyrus (PMd)		-34	-8	62
	4.98	L Postcentral Gyrus (2)	L Area 2	-36	-40	58
	3.51	N/A (PMd)		-26	-6	50
1677	8.13	L MCC (SMA)		-4	-10	50
	4.78	L MCC (24)		-6	8	40
1520	8.13	L Insula Lobe (48)		-46	-2	6
	7.42	L putamen		-26	-8	0
	7.30	L IFG (p. Opercularis) (PMv)	L Area 44	-56	6	28
1423	8.13	N/A (37)		22	-54	-26
	6.66	Cerebellar vermis (IV-V)	R Lobule I IV (Hem)	2	-54	-4
	6.16	Cerebellar vermis (VI)	R Lobule V (Hem)	6	-64	-18
	4.48	R Cerebellum (VI)	R Lobule VI (Hem)	24	-66	-22
1014	7.23	R Insula Lobe (PMv)		46	4	8
	5.84	R IFG (p. Opercularis) (PMv)	R Area 44	56	8	30
	4.91	R IFG (p. Opercularis) (PMv)	R Area 44	60	12	14
941	5.87	R Precentral Gyrus (M1)		38	-22	54
	4.42	N/A (40)	R Area hIP2 (IPS)	44	-40	42
	4.20	R Postcentral Gyrus (2)	R Area 2	42	-36	50
588	6.84	L Cerebellum (VI)	L Lobule VI (Hem)	-24	-54	-24
	4.01	L Cerebellum (VI)	L Lobule VI (Hem)	-20	-64	-14
398	8.13	L Thalamus	Thal: Premotor	-12	-20	4
373	4.56	R Rolandic Operculum (48)	R Area OP1 [SII]	48	-24	20
	4.29	R Postcentral Gyrus (S1)	R Area 3b	52	-22	42
	4.27	R SupraMarginal Gyrus (48)	R Area PFop (IPL)	50	-28	32
	3.39	R SupraMarginal Gyrus (40)	R Area PFt (IPL)	58	-32	42
	3.28	R SupraMarginal Gyrus (40)	R Area PFt (IPL)	54	-32	42
218	7.45	R Thalamus	Thal: Prefrontal	14	-18	6

Table S2: Meta-analysis of Motor Imagery (n=134)

Cluster Voxels	Z-score	Macroanatomical Location	Cytoarchitectonic/ Tractographic Label	MNI Coordinates		
				x	y	z
7104	8.13	L IFG (p. Opercularis) (PMv)	L Area 44	-52	6	10
	7.59	N/A (48)	L Area 44	-56	10	4
	6.97	L Precentral Gyrus (PMd)		-40	-6	46
	6.37	L Insula Lobe (48)		-30	18	6
	4.28	R MCC (32)		10	8	38
3554	8.13	N/A (40)	L Area hIP1 (IPS)	-36	-42	40
	7.33	L Superior Temporal Gyrus (48)	L Area PFcm (IPL)	-54	-36	24
	7.25	L SupraMarginal Gyrus (2)	L Area PFt (IPL)	-52	-34	38
2598	8.13	N/A (PMd)		32	-6	52
	7.35	R Insula Lobe (48)		34	20	6
	7.34	R Insula Lobe (48)		36	18	4
	6.40	R Precentral gyrus (PMd)		50	4	46
	6.28	R Precentral gyrus (PMv)		48	4	38
	5.87	R Putamen		22	4	6
	5.78	R IFG (p. Opercularis) (PMv)	R Area 44	56	10	10
	5.29	R IFG (p. Opercularis) (PMv)		48	8	28
	4.68	R IFG (p. Opercularis) (PMv)	R Area 44	58	10	22
	4.66	R Caudate Nucleus		18	10	10
	4.63	R Insula Lobe (48)		48	12	4
1233	8.04	R SupraMarginal Gyrus (40)	R Area 2	40	-38	46
	5.22	R SupraMarginal Gyrus (40)	R Area PF (IPL)	62	-36	30
	4.33	R Postcentral Gyrus (S1)	R Area PFt (IPL)	50	-26	40
	3.82	R Postcentral Gyrus (S1)	R Area 2	48	-32	56
597	5.03	R Superior Parietal Lobule (7)		20	-60	58
	5.00	R Precuneus (7)		12	-68	50
524	7.90	R Cerebelum (Crus 1)	R Lobule VI (Hem)	34	-56	-30
361	6.16	N/A	L Lobule VI (Hem)	-32	-56	-32
	6.01	L Cerebelum (VI)	L Lobule VI (Hem)	-30	-58	-28
223	4.26	L Middle Frontal Gyrus (DLPFC)		-36	40	22
	4.24	L Middle Frontal Gyrus (DLPFC)		-38	34	30
	4.07	L Middle Frontal Gyrus (DLPFC)		-34	44	26
	3.94	L IFG (p. Triangularis) (45)		-42	28	26

Table S3: Meta-analysis of Working Memory (n=492)

Cluster Voxels	Z-score	Macroanatomical Location	Cytoarchitectonic/ Tractographic Label	MNI Coordinates x	y	z
11113	8.13	L IFG (p. Orbitalis) (47)		-34	20	-10
	7.75	L Putamen		-18	6	4
	6.96	L Putamen		-20	4	10
	5.64	N/A		-14	8	6
	4.62	L IFG (p. Triangularis) (48)		-36	20	18
	4.37	N/A		-12	8	2
6637	8.13	R IFG (p. Orbitalis) (47)		34	22	-12
	7.24	R Middle Frontal Gyrus (PMd)		40	4	42
	6.21	N/A		16	0	14
	5.43	R Thalamus	R Thal: Prefrontal	12	-14	10
	5.13	R Middle Frontal Gyrus (PMd)		34	52	10
	3.61	R Caudate Nucleus		20	16	8
3880	8.13	N/A (19)		-26	-68	34
3869	8.13	N/A (7)		34	-58	34
	5.56	R Middle Occipital Gyrus (19)		32	-76	34
1380	8.13	L Cerebelum (Crus 1)	L Lobule VIIa crusI (Hem)	-32	-64	-32
	4.31	L Fusiform Gyrus (19)	L Area hOc4v [V4(v)]	-34	-82	-8
845	8.13	R Cerebelum (Crus 1)	R Lobule VIIa crusI (Hem)	32	-64	-32

Section 2: Conjunction analyses

Table S4: Conjunction between Motor Imagery and Movement Execution

Cluster Voxels	Z-score	Macroanatomical Location	Cytoarchitectonic/ Tractographic Label	MNI Coordinates x	y	z
1279	8.13	L MCC (SMA)		0	0	50
	4.78	L MCC (24)		-6	8	40
736	5.55	L Superior Temporal Gyrus (48)	L Area PFcm (IPL)	-50	-36	22
	5.35	L Postcentral Gyrus (S1)	L Area 3b	-40	-36	56
	5.34	L Inferior Parietal Lobule (S1)	L Area 2	-40	-34	48
	5.15	L Postcentral Gyrus (S1)	L Area 2	-38	-36	50
	4.98	L Postcentral Gyrus (2)	L Area 2	-36	-40	58
	4.90	L SupraMarginal Gyrus (S1)	L Area PFt (IPL)	-52	-28	32
	4.85	L Inferior Parietal Lobule (S1)	L Area 2	-48	-30	44
	4.75	L Inferior Parietal Lobule (S1)	L Area 2	-52	-28	42
	4.70	L SupraMarginal Gyrus (48)	L Area OP1 [SII]	-52	-30	28
729	7.30	L IFG (p. Opercularis) (PMv)	L Area 44	-56	6	28
	7.27	L IFG (p. Opercularis) (PMv)		-50	4	10
442	5.00	R IFG (p. Opercularis) (PMv)	R Area 44	54	8	10
	4.95	R IFG (p. Opercularis) (PMv)		52	8	30
	4.87	R IFG (p. Opercularis) (PMv)		52	6	34
	4.68	R IFG (p. Opercularis) (PMv)	R Area 44	58	10	22
	4.65	R IFG (p. Opercularis) (PMv)	R Area 44	58	12	14
290	6.42	L Putamen		-26	0	4
246	4.42	N/A (40)	R Area hIP2 (IPS)	44	-40	42
	4.20	R Postcentral Gyrus (2)	R Area 2	42	-36	50
	3.77	R Postcentral Gyrus (S1)	R Area 2	46	-32	56
223	5.03	L Precentral Gyrus (PMd)		-34	-8	62
	4.26	L Precentral Gyrus (M1)		-40	-12	54
	3.51	N/A (PMd)		-26	-6	50
153	4.97	L Cerebellum (VI)	L Lobule VI (Hem)	-28	-58	-24
152	5.64	R Cerebelum (VI)	R Lobule VI (Hem)	32	-54	-24
132	4.66	R Middle Frontal Gyrus (pre-SMA)		36	-8	62
64	3.96	R Postcentral Gyrus (S1)	R Area 3b	52	-24	40
	3.28	R SupraMarginal Gyrus (40)	R Area PFt (IPL)	54	-32	42
	3.25	R SupraMarginal Gyrus (40)	R Area PFt (IPL)	58	-32	44

Table S5: Conjunction between Motor Imagery and Working Memory

Cluster Voxels	Z-score	Macroanatomical Location	Cytoarchitectonic/ Tractographic Label	MNI Coordinates x	y	z
4389	8.13	L IFG (p. Opercularis) (PMv)	L Area 44	-54	6	30
	7.65	L IFG (p. Opercularis) (PMv)	L Area 44	-54	10	16
	6.97	L Precentral Gyrus (PMd)		-40	-6	46
	6.37	L Insula Lobe (48)		-30	18	6
	6.27	L Putamen		-20	0	10
	5.70	L Putamen		-22	6	4
	3.83	L IFG (p. Orbitalis)		-48	16	-2
1933	8.13	N/A (40)	L Area hIP1 (IPS)	-36	-42	40
954	8.13	N/A (PMd)		30	-6	52
	5.99	R Precentral Gyrus (PMv)		46	4	38
	5.29	R IFG (p. Opercularis) (PMv)		48	8	28
	4.98	R Middle Frontal Gyrus (PMd)		44	4	44
	3.93	R IFG (p. Opercularis) (PMv)	R Area 45	54	12	18
	3.90	R IFG (p. Opercularis) (PMv)	R Area 45	54	14	14
682	8.04	R SupraMarginal Gyrus (40)	R Area 2	40	-38	46
	4.07	R Postcentral Gyrus (S1)	R Area PFt (IPL)	50	-28	44
547	5.03	R Superior Parietal Lobule (7)		20	-60	58
	5.00	R Precuneus (7)		12	-68	50
371	7.80	R Cerebelum (Crus 1)	R Lobule VIIa crusI (Hem)	34	-58	-30
324	7.35	R Insula Lobe		34	20	6
	4.04	R Caudate Nucleus		16	10	8
	3.98	N/A		18	8	12
	3.49	R Putamen (48)		22	16	4
	3.45	R Caudate Nucleus		20	16	8
219	4.26	L Middle Frontal Gyrus (DLPFC)		-36	40	22
	4.24	L Middle Frontal Gyrus (DLPFC)		-38	34	30
	4.07	L Middle Frontal Gyrus (DLPFC)		-34	44	26
	3.94	L IFG (p. Triangularis) (45)		-42	28	26
203	5.94	L Cerebelum (VI)	L Lobule VI (Hem)	-32	-56	-30

Table S6: Conjunction between Movement Execution and Working Memory

Cluster Voxels	Z-score	Macroanatomical Location	Cytoarchitectonic/ Tractographic Label	MNI Coordinates x	y	z
828	8.13	L MCC (SMA)		-2	-2	50
	4.78	L MCC (24)		-6	8	40
373	7.07	L IFG (p. Opercularis) (PMv)	L Area 44	-54	6	28
	5.26	L IFG (p. Opercularis) (PMv)	L Area 44	-54	6	16
	4.36	L IFG (p. Opercularis) (pre-SMA)	L Area 44	-48	10	6
298	5.88	L Postcentral Gyrus (S1)	L Area 3a	-38	-34	48
	4.00	L Inferior Parietal Lobule (40)	L Area 2	-38	-42	56
251	5.78	R Cerebelum (VI)	R Lobule VI (Hem)	28	-56	-24
	4.48	R Cerebelum (VI)	R Lobule VI (Hem)	24	-66	-22
210	4.42	N/A (40)	R Area hIP2 (IPS)	44	-40	42
	4.20	R Postcentral Gyrus (2)	R Area 2	42	-36	50
154	7.32	L Thalamus	Thal : Prefrontal	-12	-16	8
150	5.31	R IFG (p. Opercularis) (PMv)		52	8	32
	3.65	R IFG (p. Opercularis) (PMv)	R Area 44	54	12	16
	3.63	R IFG (p. Opercularis) (PMv)	R Area 45	56	14	18
129	5.43	R Thalamus	R Thal: Prefrontal	12	-14	10
120	4.86	L Precentral Gyrus (PMd)		-34	-8	60
	3.51	N/A (PMd)		-26	-6	50
79	4.39	L Cerebelum (VI)		-30	-56	-28
54	4.78	L Putamen		-22	2	4
	4.38	L Putamen		-24	6	4
	3.89	L Pallidum		-20	-2	6
48	3.86	R Middle Frontal Gyrus (PMd)		34	-6	60
7	3.28	R SupraMarginal Gyrus (40)	R Area PFt (IPL)	54	-32	42

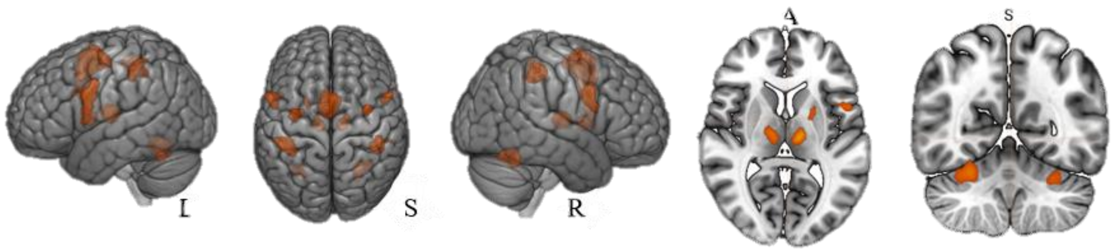

Fig. S1. Conjunction between Movement Execution and Working Memory

Table S7: Conjunction between Movement Execution, Motor Imagery and Working Memory

Cluster Voxels	Z-score	Macroanatomical Location	Cytoarchitectonic/ Tractographic Label	MNI Coordinates x	y	z
790	8.13	L MCC (SMA)		0	0	50
	4.78	L MCC (24)		-6	8	40
373	7.07	L IFG (p. Opercularis) (PMv)	L Area 44	-54	6	28
	5.26	L IFG (p. Opercularis) (PMv)	L Area 44	-54	6	16
	4.36	L IFG (p. Opercularis) (pre-SMA)	L Area 44	-48	10	6
278	5.34	L Inferior Parietal Lobule (S1)	L Area 2	-40	-34	48
	4.00	L Inferior Parietal Lobule (40)	L Area 2	-38	-42	56
202	4.42	N/A (40)	R Area hIP2 (IPS)	44	-40	42
	4.20	R Postcentral Gyrus (2)	R Area 2	42	-36	50
150	4.95	R IFG (p. Opercularis) (PMv)		52	8	30
	4.87	R IFG (p. Opercularis) (PMv)		52	6	34
	3.65	R IFG (p. Opercularis) (PMv)	R Area 44	54	12	16
130	5.30	R Cerebelum (VI)	R Lobule VI (Hem)	32	-56	-24
120	4.86	L Precentral Gyrus (PMd)		-34	-8	60
	3.51	N/A (PMd)		-26	-6	50
75	4.39	L Cerebelum (VI)		-30	-56	-28
54	4.78	L Putamen		-22	2	4
	4.38	L Putamen		-24	6	4
	3.89	L Pallidum		-20	-2	6
48	3.86	R Middle Frontal Gyrus (PMd)		34	-6	60
7	3.28	R SupraMarginal Gyrus (40)	R Area PFt (IPL)	54	-32	42

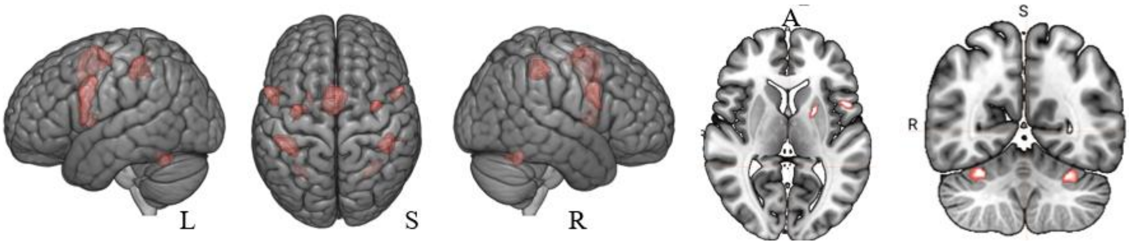

Fig. S2. Conjunction between motor imagery, movement execution and working memory
In the main analysis we talked about overlapping by counting the motor imagery network as a reference. Now, if we take movement execution as a reference point. Movement execution shares 39% of its total volume with motor imagery and 24% with working memory. Then, if we take working memory as a reference, it shares 34% of its networks with motor imagery while it shares only 9% with movement execution.

<u>*Discussion about conjunction between all paradigms:*</u>

Our conjunction analysis revealed that movement execution, motor imagery, and working memory consistently recruited a shared network including the supplementary motor area (SMA

and pre-SMA), primary somatosensory cortex (S1), dorsal and ventral premotor cortices (PMd, PMv), and lobule VI of the cerebellum.

The SMA and pre-SMA are central to motor initiation and sequential planning, with the SMA proper more directly related to execution and the pre-SMA implicated in higher-order action selection and cognitive control (Hétu et al., 2013; Nachev et al., 2008). Their common activation across paradigms likely reflects shared demands for internally generated movement planning and temporal organization.

For the premotor cortices, PMd is classically engaged in the integration of sensory information for action selection and motor preparation, while PMv contributes to sensorimotor integration and the representation of goal-directed actions (Hoshi & Tanji, 2007; Rizzolatti & Craighero, 2004). Their recruitment across execution, imagery, and working memory suggests that each paradigm involves maintaining and manipulating motor representations that require premotor computations.

Activation of S1 is consistent with the idea that both overt and covert action involve the (re)activation of expected sensory consequences. Even in the absence of overt movement, motor imagery and working memory appear to elicit somatosensory predictions that ground motor representations in embodied feedback (Kilteni et al., 2018; Naito et al., 2002).

Finally, cerebellar lobule VI was recruited in all three paradigms. While classically associated with fine motor coordination and sensorimotor prediction, lobule VI is also increasingly recognized as part of an integrative "somato-cognitive action network" (Buckner et al., 2011; Gordon et al., 2023). Its consistent involvement may reflect the cerebellum's dual contribution to both motor control and executive processes, enabling the generation and refinement of internal models during execution, imagery, and working memory (McDougle et al., 2022; Ramnani, 2006).

Section 3: Contrast analyses

Methods:

Random effects ALE subtraction analysis was employed to compare the resulting meta-analyses (Eickhoff et al., 2012). First, voxel-wise differences between ALE maps were computed for each pool of experiments. Subsequently, experiments were randomly shuffled into two equal-sized samples, and voxel-wise differences between their ALE scores were recorded. This shuffling process was iterated 10,000 times to generate an empirical null distribution of ALE score differences. The map of differences was thresholded at a posterior probability for true differences of $P > 0.95$ and inclusively masked by the respective main effect of the minuend (Chase et al., 2011; Rottschy et al., 2012) with a minimum cluster volume of 100 mm^3 (Beissner et al., 2013; Erickson et al., 2014; Turkeltaub et al., 2012).

Table S8: Motor Imagery vs Movement Execution

Cluster Voxels	Z-score	Macroanatomical Location	Cytoarchitectonic/ Tractographic Label	MNI Coordinates x	y	z
1734	8.13	N/A (PMd)		-22	-12	52
	6.04	L Precentral Gyrus (PMv)		-40	-4	42
	5.78	L Precentral Gyrus (PMd)		-36	-4	44
	3.35	L Precentral Gyrus (PMd)		-48	0	52
	2.81	L Precentral Gyrus (PMd)		-36	-4	52
	2.76	L Precentral Gyrus (PMd)		-38	-2	54
	2.75	R Posterior-Medial Frontal (pre-SMA)		10	4	68
	2.67	R Posterior-Medial Frontal (SMA)		10	0	70
	2.03	L IFG (p. Triangularis) (PMv)		-48	12	36
	1.95	L IFG (p. Opercularis) (PMv)	L Area 44	-48	6	34
1274	8.13	N/A (40)	L Area hIP2 (IPS)	-40	-42	40
	7.65	L Inferior Parietal Lobule (40)	L Area hIP3 (IPS)	-40	-48	52
	7.29	L Superior Parietal Lobule (7)		-20	-64	54
	3.35	L Inferior Parietal Lobule (7)	L Area 7PC (SPL)	-32	-58	58
	3.12	L Superior Parietal Lobule	L Area 7A (SPL)	-18	-74	58
	2.71	L Inferior Parietal Lobule (40)	L Area hIP1 (IPS)	-36	-54	50
	2.69	L Precuneus (7)		-14	-64	42
	2.42	N/A (5)		-18	-50	60
	2.30	L SupraMarginal Gyrus (40)	L Area PF (IPL)	-52	-38	34
	1.99	L SupraMarginal Gyrus		-64	-36	40
484	6.14	N/A (PMd)		26	-4	50
	6.09	N/A (PMd)		30	-2	50
	5.63	N/A (PMd)		30	-6	48
	3.72	N/A (PMd)		32	-8	50
	2.26	R IFG (p. Opercularis) (PMd)		44	8	42
	2.24	R IFG (p. Opercularis) (PMd)		46	6	44

	1.90	R Middle Frontal Gyrus (PMd)	R Area 44	50	6	54
253	3.35	L Temporal Pole		-46	14	-8
	3.19	L Temporal Pole		-50	16	-6
	3.01	N/A		-56	16	0
	2.99	N/A (48)		-62	8	4
191	3.40	L Insula Lobe (48)		-26	20	10
	2.05	L Insula Lobe (48)		-38	14	2
160	2.91	R Precuneus (7)		10	-72	46
	2.75	R Superior Occipital Gyrus (7)		22	-72	42
104	3.01	L Superior Medial Gyrus (pre-SMA)		2	20	48
98	2.18	L Middle Frontal Gyrus		36	-56	-36
	2.01	L Middle Frontal Gyrus		30	-68	-30
	1.79	L IFG (p. Triangularis)		38	-64	-28
64	2.25	L Superior Temporal Gyrus (48)	L Area PFop (IPL)	-62	-32	24
	1.71	L Superior Temporal Gyrus (42)		-58	-42	22
38	2.50	R Insula Lobe (47)		42	22	2
32	2.29	R Superior Parietal Lobule (7)	R Area 7A (SPL)	14	-66	62
29	2.04	L Cerebelum (Crus 1)	L Lobule VI (Hem)	-36	-50	-32
20	2.08	R MCC		12	6	40
14	1.84	N/A (40)		38	-32	40
	1.83	R Postcentral Gyrus (40)	R Area 3a	36	-34	44

Table S9: Movement Execution vs Motor Imagery

Cluster Voxels	Z-score	Macroanatomical Location	Cytoarchitectonic/ Tractographic Label	MNI Coordinates		
				x	y	z
1256	8.13	L MCC (SMA)		0	0	50
	4.66	L MCC (24)		-6	10	42
984	6.78	L IFG (p. Opercularis) (PMv)	L Area 44	-50	6	8
	6.62	L IFG (p. Opercularis) (PMv)	L Area 44	-56	6	26
	6.44	L IFG (p. Opercularis) (PMv)	L Area 44	-54	8	28
	6.09	L Putamen		-24	0	4
	5.97	L IFG (p. Opercularis) (PMv)	L Area 44	-56	6	32
	5.90	L Pallidum		-22	-4	4
	3.80	L Insula Lobe (48)		-42	8	6
	3.72	L Insula Lobe (48)		-44	8	2
725	5.15	L Postcentral Gyrus (S1)	L Area 2	-38	-36	50
	4.98	L Postcentral Gyrus (2)	L Area 2	-36	-40	58
	4.91	L Inferior Parietal Lobule (S1)	L Area 2	-40	-34	46
	4.65	L Postcentral Gyrus (M1)	L Area 2	-42	-38	56
	4.62	L Inferior Parietal Lobule (48)	L Area 2	-44	-34	48
	4.58	L Superior Temporal Gyrus (48)	L Area OP1 [SII]	-52	-34	24

	4.15	L SupraMarginal Gyrus (2)	L Area PFop (IPL)	-54	-30	32
	4.03	L SupraMarginal Gyrus (S1)	L Area PFt (IPL)	-52	-28	38
	1.95	L Postcentral Gyrus (48)	L Area OP1 [SII]	-60	-24	20
447	4.10	R IFG (p. Opercularis) (PMv)	R Area 44	56	10	10
	4.04	R Precentral Gyrus (PMv)		52	6	36
	3.67	R IFG (p. Opercularis) (PMv)	R Area 44	52	12	10
	3.66	R IFG (p. Opercularis) (PMv)		48	6	34
	3.50	R IFG (p. Opercularis) (PMv)	R Area 44	52	10	26
	3.35	R IFG (p. Triangularis) (PMv)		50	10	30
	3.01	R Insula Lobe (48)		44	12	4
	2.97	R IFG (p. Opercularis) (48)		48	14	6
	2.93	R IFG (p. Opercularis) (S1)		46	12	8
280	4.40	R SupraMarginal Gyrus (40)	R Area PFt (IPL)	44	-38	44
	4.14	R Postcentral Gyrus (M1)	R Area 2	40	-36	50
	2.33	R Postcentral Gyrus (S1)	R Area 2	48	-32	56
226	5.03	L Precentral Gyrus (PMd)		-34	-8	62
	3.55	L Precentral Gyrus (M1)		-40	-12	52
	3.51	N/A (PMd)		-26	-6	50
	3.33	L Precentral Gyrus (M1)		-30	-14	64
	3.28	L Precentral Gyrus (M1)		-44	-12	54
	3.24	L Precentral Gyrus (M1)		-30	-18	54
160	4.27	L Cerebelum (VI)	L Lobule VI (Hem)	-28	-58	-28
	2.97	L Cerebelum (VI)	L Lobule VI (Hem)	-28	-62	-20
152	5.35	R Cerebelum (VI)	R Lobule VI (Hem)	34	-54	-26
137	4.23	R Middle Frontal Gyrus (pre-SMA)		36	-8	60
81	3.01	R Postcentral Gyrus (S1)	R Area PFt (IPL)	50	-26	38
	2.81	R SupraMarginal Gyrus (40)	R Area PFt (IPL)	52	-32	44
45	3.11	L Cerebelum (IV-V)	L Lobule I IV (Hem)	-4	-52	-8
28	2.61	R Thalamus	R Thal: Prefrontal	12	-10	4
	2.44	N/A		16	-14	2
6	1.90	N/A	L Thal: Prefrontal	-14	-10	8
3	1.90	R Cerebelum (VI)	R Lobule VI (Hem)	8	-68	-16

Table S10: Motor Imagery vs Working Memory

Cluster Voxels	Z-score	Macroanatomical Location	Cytoarchitectonic/ Tractographic Label	MNI Coordinates		
				x	y	z
2215	8.13	N/A (PMd)		-20	-10	52
	5.71	L Superior Frontal Gyrus (PMd)		-20	2	66
	3.24	R MCC (pre-SMA)		10	2	50
	2.99	L Precentral Gyrus (M1)		-44	-10	56
	2.29	N/A		12	4	38
	2.02	N/A		-22	-8	44

1011	7.33	L Superior Temporal Gyrus (48)	L Area PFcm (IPL)	-54	-36	24
	7.25	L SupraMarginal Gyrus (2)	L Area PFt (IPL)	-52	-34	38
833	8.13	L IFG (p. Opercularis) (PMv)	L Area 44	-52	6	10
	6.58	L Temporal Pole (48)		-52	8	0
	3.54	L IFG (p. Opercularis) (PMv)	L Area 44	-60	4	24
	2.79	L Precentral Gyrus (PMv)	L Area 44	-58	2	30
	2.12	L Temporal Pole (48)		-42	10	-10
690	8.13	R Middle Frontal Gyrus (PMd)		32	-8	58
	6.23	R Precentral Gyrus (PMd)		48	4	48
	3.04	R Precentral Gyrus (PMv)		54	4	40
	2.99	R Precentral Gyrus (M1)		42	-10	52
608	8.13	L Inferior Parietal Lobule (40)	L Area 2	-38	-42	56
	3.72	L Superior Parietal Lobule (5)		-16	-58	56
	3.54	N/A		-16	-56	52
	2.60	L Postcentral Gyrus (40)		-34	-38	48
	2.37	L Postcentral Gyrus (7)	L Area 5L (SPL)	-26	-48	66
	1.82	L Superior Parietal Lobule (7)	L Area 7A (SPL)	-26	-64	64
279	7.39	L Pallidum		-22	-2	8
261	4.57	R IFG (p. Opercularis) (PMv)	R Area 44	56	8	14
	4.28	R IFG (p. Opercularis) (PMv)		50	8	6
	3.72	R Rolandic Operculum (PMv)	R Area 44	54	6	8
	2.99	R IFG (p. Opercularis) (40)	R Area 44	62	10	26
	2.93	R IFG (p. Opercularis) (PMv)	R Area 44	60	8	22
260	5.22	R SupraMarginal Gyrus (40)	R Area PF (IPL)	62	-36	30
	3.35	R SupraMarginal Gyrus (40)	R Area PF (IPL)	58	-40	44
222	5.87	R Putamen		22	4	6
165	4.16	R Cerebelum (Crus 1)	R Lobule VIIa crusI (Hem)	44	-54	-32
	3.72	R Cerebelum (Crus 2)	R Lobule VIIa crusI (Hem)	40	-54	-34
	3.54	R Cerebelum (Crus 2)	R Lobule VIIa crusI (Hem)	40	-50	-34
127	2.66	N/A (M1)		34	-30	42
	2.35	R Postcentral Gyrus (2)	R Area 2	34	-42	58
	2.30	R Postcentral Gyrus (S1)	R Area 4p	38	-34	54
50	3.54	R Postcentral Gyrus (S1)	R Area 3b	52	-22	36
21	2.00	N/A		-24	-56	-30
	1.80	L Cerebelum (VI)	L Lobule VI (Hem)	-28	-58	-24
	1.73	L Cerebelum (VI)	L Lobule VI (Hem)	-26	-60	-18
17	1.92	L Cerebelum (Crus 1)	L Lobule VIIa crusI (Hem)	-36	-52	-32
8	2.01	R Postcentral Gyrus (S1)	R Area 1	46	-28	56
5	1.76	N/A (5)	R Area 7PC (SPL)	20	-54	58
	1.73	N/A (7)	R Area 7PC (SPL)	24	-52	58

Table S11: Working Memory vs Motor Imagery

Cluster Voxels	Z-score	Macroanatomical Location	Cytoarchitectonic/ Tractographic Label	MNI Coordinates x	y	z
3693	8.13	R Middle Frontal Gyrus (45)		40	46	18
	5.52	R Middle Frontal Gyrus (PMd)		36	4	42
	5.08	R Middle Frontal Gyrus (10)		34	50	12
	3.72	R IFG (p. Opercularis) (PMv)		38	10	38
3661	8.13	L IFG (p. Orbitalis) (47)		-32	24	-10
	7.82	L Insula Lobe (48)		-32	18	16
	5.38	L Middle Orbital Gyrus (47)	L Area Fp1	-36	54	0
	4.62	L IFG (p. Triangularis) (48)		-36	20	18
	3.72	L IFG (p. Triangularis) (48)		-30	30	10
	2.77	L IFG (p. Triangularis) (45)		-46	20	8
2117	8.13	N/A (7)		34	-58	34
	5.90	R Middle Occipital Gyrus (7)		32	-72	38
	5.78	N/A (19)		34	-68	36
	3.72	R Middle Occipital Gyrus (19)		32	-74	32
	3.35	R Middle Occipital Gyrus (19)		34	-78	36
1512	8.13	N/A (19)		-26	-68	34
	5.81	N/A (19)		-28	-80	22
	5.17	L Middle Occipital Gyrus (19)		-30	-80	28
	3.19	N/A (19)		-26	-70	22
	2.95	L Inferior Parietal Lobule (40)	L Area hIP2 (IPS)	-48	-46	42
	2.88	L Inferior Parietal Lobule (40)	L Area hIP2 (IPS)	-48	-46	48
1312	8.13	R MCC (32)		8	22	36
	4.93	L ACC (32)		-8	30	28
	4.01	L ACC (24)		-6	24	28
	3.72	R ACC (32)		12	26	30
	2.49	L MCC (24)		0	14	38
882	6.94	L Fusiform Gyrus (19)	L Area FG2	-42	-66	-10
	6.28	L Fusiform Gyrus (37)	L Area FG4	-40	-62	-12
	4.60	L Cerebelum (Crus 1)	L Area FG2	-40	-74	-14
	3.72	L Fusiform Gyrus (19)	L Area FG2	-42	-72	-12
	3.54	L Cerebelum (Crus 1)	L Lobule VIIa crusI (Hem)	-40	-64	-20
	3.43	L Inferior Occipital Gyrus (19)	L Area FG1	-34	-80	-4
	3.24	L Cerebelum (Crus 1)	L Lobule VIIa crusI (Hem)	-38	-68	-22
	3.16	L Cerebelum (Crus 1)	L Lobule VIIa crusI (Hem)	-38	-72	-22
	2.88	L Cerebelum (Crus 1)		-28	-68	-32
681	8.13	R IFG (p. Orbitalis) (47)		34	22	-12
	7.48	N/A (48)		30	16	14
	3.72	N/A (48)		28	18	12
	2.29	R Insula Lobe (48)		38	26	12
	2.23	R Putamen (48)		26	14	8
415	8.13	L Thalamus	L Thal: Prefrontal	-10	-12	10
	3.12	N/A		-12	6	6
	2.93	L Putamen (48)		-16	6	-2
	2.78	L Putamen (48)		-14	8	-4

393	6.14	N/A		14	-2	14
	3.54	R Thalamus	R Thal: Temporal	4	-18	12
	3.40	R Thalamus	R Thal: Temporal	2	-16	10
240	3.55	R Cerebelum (VI)	R Lobule VI (Hem)	28	-62	-14
	2.88	R Cerebelum (VI)	R Lobule VI (Hem)	24	-70	-20
	1.77	R Cerebelum (Crus 1)	R Lobule VIIa crusI (Hem)	38	-68	-30
54	2.20	L Precuneus (7)		2	-68	52
19	2.07	L Superior Parietal Lobule (7)		-12	-74	46
	1.78	L Precuneus (7)		-4	-68	50

Table S12: Movement Execution vs Working Memory

Cluster Voxels	Z-score	Macroanatomical Location	Cytoarchitectonic/ Tractographic Label	MNI Coordinates		
				x	y	z
2768	8.13	L Postcentral Gyrus (S1)	L Area 3b	-38	-30	50
	6.60	L Rolandic Operculum (48)	L Area OP1 [SII]	-46	-26	18
	5.31	L SupraMarginal Gyrus (48)	L Area OP1 [SII]	-52	-26	28
	4.98	L Precentral Gyrus (PMd)		-34	-10	64
	4.94	L Postcentral Gyrus	L Area 3b	-36	-40	60
	3.72	L Insula Lobe (48)	L Area OP3 [VS]	-38	-18	20
	3.44	L SupraMarginal Gyrus (48)	L Area PFop (IPL)	-58	-34	30
1270	8.13	N/A (37)		22	-54	-26
	6.66	Cerebellar Vermis (4/5)	R Lobule I IV (Hem)	2	-54	-4
	6.16	Cerebellar Vermis (6)	R Lobule I IV (Hem)	6	-64	-18
	3.16	N/A (18)		-6	-56	-16
1237	8.13	L MCC (SMA)		-4	-10	50
	2.57	L MCC (24)		-8	4	38
1206	8.13	L Insula Lobe (48)		-46	-2	6
	7.42	L Putamen		-26	-8	0
	3.72	L Insula Lobe (48)		-38	-4	14
	3.35	L Precentral Gyrus (PMv)	L Area 44	-58	4	28
883	7.23	R Insula Lobe (PMv)		46	4	8
	5.75	R IFG (p. Opercularis) (PMv)	R Area 44	56	6	30
	4.91	R IFG (p. Opercularis) (PMv)	R Area 44	60	12	14
670	5.87	R Precentral Gyrus (M1)		38	-22	54
508	6.84	L Cerebelum (VI)	L Lobule VI (Hem)	-24	-54	-24
	3.72	L Cerebelum (VI)	L Lobule VI (Hem)	-22	-56	-18
	3.54	L Cerebelum (VI)	L Lobule VI (Hem)	-20	-62	-16
363	4.49	R Rolandic Operculum (48)	R Area OP1 [SII]	50	-24	20
	4.09	R Postcentral Gyrus (S1)	R Area 3b	54	-22	40
	3.72	R Rolandic Operculum (48)	R Area OP1 [SII]	50	-28	28
	3.43	R Postcentral Gyrus (S1)	R Area 4p	50	-20	42
332	8.13	L Thalamus	L Thal: Premotor	-12	-20	4

153	7.45	R Thalamus	R Thal: Prefrontal	14	-18	6

Table S13: Working Memory vs Movement Execution

Cluster Voxels	Z-score	Macroanatomical Location	Cytoarchitectonic/ Tractographic Label	MNI Coordinates		
				x	y	z
7134	8.13	L IFG (p. Orbitalis) (47)		-34	18	-8
	6.41	L Precentral Gyrus (PMd)		-46	0	52
	4.35	L IFG (p. Orbitalis) (47)		-38	24	-12
	3.54	L Middle Frontal Gyrus (45)		-46	40	28
	3.50	L ACC (32)		-12	30	32
	2.89	L IFG (p. Triangularis) (48)		-36	20	18
	1.96	N/A (PMd)		-24	-14	48
5192	8.13	R IFG (p. Orbitalis) (47)		34	24	-12
	4.82	R Middle Frontal Gyrus (10)	R Area Fp1	34	52	8
	4.41	R Middle Frontal Gyrus (DLPFC)		38	52	10
	4.16	R Middle Frontal Gyrus (45)		50	44	16
	3.75	R IFG (p. Triangularis) (48)		46	18	18
	3.72	R IFG (p. Triangularis) (45)		48	36	12
	3.54	R Middle Frontal Gyrus (DLPFC)		42	48	12
	3.54	R Middle Frontal Gyrus (45)		40	46	14
	3.43	R Middle Frontal Gyrus (45)		38	48	18
2926	8.13	N/A (7)		34	-58	34
	5.56	R Middle Occipital Gyrus (19)		32	-76	34
	4.15	R Precuneus (7)		8	-72	44
	3.72	R Precuneus (7)		6	-68	44
	3.54	R Precuneus (7)		8	-68	48
	3.41	R Superior Parietal Lobule (7)	R Area 7A (SPL)	16	-68	64
	3.35	R Superior Parietal Lobule (7)	R Area 7A (SPL)	20	-68	62
	3.04	R Precuneus (7)		16	-66	50
2780	8.13	N/A (19)		-26	-68	34
	3.72	L Superior Parietal Lobule (7)	L Area hIP3 (IPS)	-28	-66	58
479	3.43	L Inferior Temporal Gyrus (20)		-52	-52	-12
	3.24	L Fusiform Gyrus (37)	L Area FG4	-42	-58	-14
	3.12	L Inferior Temporal Gyrus (37)	L Area FG4	-48	-58	-14
	2.99	L Fusiform Gyrus	L Area FG3	-36	-58	-8
	2.45	L Cerebelum (Crus 1)	L Lobule VIIa crusI (Hem)	-36	-70	-30
	2.44	L Cerebelum (Crus 1)	L Lobule VIIa crusI (Hem)	-42	-68	-20
	1.86	L Fusiform Gyrus (19)	L Area FG2	-36	-70	-10
238	3.16	R Cerebelum (Crus 1)	R Lobule VIIa crusI (Hem)	32	-68	-32
	3.01	R Cerebelum (Crus 1)	R Lobule VIIa crusI (Hem)	36	-64	-24
	1.95	R Cerebelum (VI)	R Area FG1	32	-64	-14
222	8.13	N/A		-14	2	12
	2.32	L Thalamus	L Thal: Temporal	-2	-14	8
	2.29	L Thalamus	L Thal: Temporal	-4	-12	12
56	2.42	R Caudate Nucleus		12	-6	22
	2.11	N/A	R Thal: Temporal	4	-8	8

	1.87	N/A	R Thal: Temporal	2	-12	8
34	2.51	L Inferior Occipital Gyrus (19)	L Area hOc4la	-36	-82	-4
20	2.58	R Caudate Nucleus		10	6	2
	2.21	R Caudate Nucleus		12	10	2
	2.02	R Caudate Nucleus		12	8	10
2	1.78	L Putamen		-18	6	6

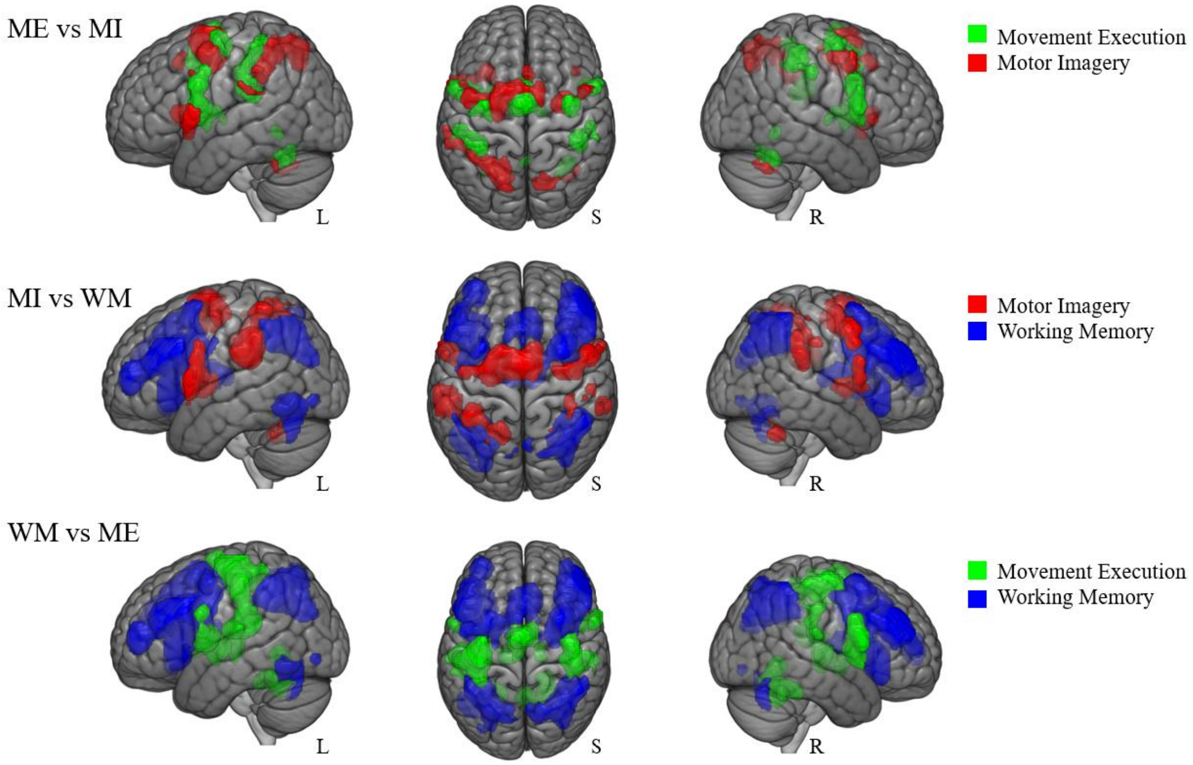

Fig. S3. Contrast analyses

Section 4: Balanced contrast analyses

Table S14: Meta-analysis of Motor Imagery vs Movement Execution

Cluster Voxels	Z-score	Macroanatomical Location	Cytoarchitectonic/ Tractographic Label	MNI Coordinates		
				x	y	z
3562	0.98	L Posterior-Medial Frontal (SMA)		-4	0	60
	0.60	L Middle Frontal Gyrus (PMd)		-26	-6	56
	0.05	L IFG (p. Opercularis) (PMv)	L Area 44	-56	8	12
	0.03	L IFG (p. Opercularis) (PMv)	L Area 44	-54	6	26
	0.01	L Precentral Gyrus (PMd)		-38	-6	46
	0.00	L Precentral Gyrus (PMv)		-46	-2	42
	0.00	L Precentral Gyrus (PMd)		-52	-4	46
1369	0.26	L Inferior Parietal Lobule (40)		-38	-40	46
	0.01	L SupraMarginal Gyrus (40)	L Area PFt (IPL)	-52	-34	40
	0.01	L Superior Parietal Lobule (7)		-18	-62	54
	0.00	L SupraMarginal Gyrus (2)	L Area PFop (IPL)	-54	-32	32
	0.00	L SupraMarginal Gyrus (48)	L Area PFop (IPL)	-56	-34	30
	0.00	L Inferior Parietal Lobule (7)	L Area hIP3 (IPS)	-28	-60	52
	0.00	L Superior Temporal Gyrus (48)	L Area PFcm (IPL)	-52	-38	22
	0.00	L Superior Parietal Lobule (7)	L Area hIP3 (IPS)	-26	-64	54
383	0.04	R Precentral Gyrus (PMd)		30	-8	54
204	0.00	R Cerebelum (Crus 1)	R Lobule VIIa crusI (Hem)	34	-60	-32
192	0.00	N/A (40)	R Area 7PC (SPL)	38	-38	42
	0.00	N/A (40)	R Area hIP2 (IPS)	42	-40	40
	0.00	N/A (40)		32	-34	42
124	0.00	R Precentral Gyrus (PMv)		48	2	34
53	0.00	L Pallidum		-22	-2	4

Table S15: Meta-analysis of Motor Imagery vs Working Memory

Cluster Voxels	Z-score	Macroanatomical Location	Cytoarchitectonic/ Tractographic Label	MNI Coordinates		
				x	y	z
6253	1.00	L Posterior-Medial Frontal (pre-SMA)		0	2	50
	0.99	L IFG (p. Opercularis) (PMv)	L Area 44	-54	8	12
	0.96	L IFG (p. Opercularis) (PMv)	L Area 44	-54	6	32
	0.80	L Precentral Gyrus (PMd)		-40	-6	46
	0.64	L Pallidum		-22	-2	6
	0.05	L Insula Lobe (48)		-32	18	8
3055	1.00	L Inferior Parietal Lobule (40)		-38	-40	44
	1.00	L Inferior Parietal Lobule (40)	L Area hIP3 (IPS)	-38	-44	46
	0.90	L Superior Parietal Lobule (7)		-16	-62	56
	0.85	L SupraMarginal Gyrus (2)	L Area PFt (IPL)	-54	-34	38
	0.82	L SupraMarginal Gyrus (48)	L Area PFop (IPL)	-56	-34	30
	0.00	L Postcentral Gyrus (7)	L Area 5L (SPL)	-28	-48	66
1930	0.98	R Precentral Gyrus (PMd)		32	-6	54
	0.40	R Precentral Gyrus (PMd)		50	2	44
	0.39	R Precentral Gyrus (PMv)		48	4	38
	0.18	R Insula Lobe (48)		38	18	2
	0.06	R IFG (p. Opercularis) (PMv)	R Area 44	54	10	10
	0.03	R IFG (p. Opercularis) (PMv)	R Area 44	56	10	22
968	0.72	R SupraMarginal Gyrus (40)	R Area 2	40	-38	46
	0.01	R Postcentral Gyrus (S1)	R Area PFt (IPL)	50	-28	44
	0.01	R Postcentral Gyrus (S1)	R Area PFt (IPL)	50	-26	40
	0.00	R Superior Temporal Gyrus (48)	R Area PFcm (IPL)	62	-36	28
463	0.61	R Cerebelum (Crus 1)	R Lobule VIIa crusI (Hem)	34	-58	-30
455	0.01	N/A (7)		20	-60	56
	0.01	R Precuneus (7)		14	-66	54
	0.01	R Precuneus (7)		12	-68	52
	0.00	N/A (7)		22	-64	48
294	0.05	N/A	L Lobule VI (Hem)	-32	-58	-32
268	0.09	R Putamen		24	2	4
	0.03	R Caudate Nucleus		18	10	10
	0.00	N/A		20	16	4
37	0.02	R Thalamus	R Thal: Prefrontal	14	-12	4
4	0.00	N/A		-14	0	14
4	0.01	N/A	L Thal: Prefrontal	-14	-10	8
2	0.00	L Pallidum		-16	-2	2

Bibliography

Beissner, F., Meissner, K., Bär, K.-J., & Napadow, V. (2013). The Autonomic Brain : An Activation Likelihood Estimation Meta-Analysis for Central Processing of Autonomic Function. *The Journal of Neuroscience*, *33*(25), 10503. https://doi.org/10.1523/JNEUROSCI.1103-13.2013

Buckner, R. L., Krienen, F. M., & Castellanos, A. (2011). The organization of the human cerebellum estimated by intrinsic functional connectivity. *Journal of Neurophysiology*, *106*(5), 2322-2345. https://doi.org/10.1152/jn.00339.2011

Chase, H. W., Eickhoff, S. B., Laird, A. R., & Hogarth, L. (2011). The Neural Basis of Drug Stimulus Processing and Craving : An Activation Likelihood Estimation Meta-Analysis. *Biological Psychiatry*, *70*(8), 785-793. https://doi.org/10.1016/j.biopsych.2011.05.025

Eickhoff, S. B., Bzdok, D., Laird, A. R., Kurth, F., & Fox, P. T. (2012). Activation likelihood estimation meta-analysis revisited. *NeuroImage*, *59*(3), 2349-2361. https://doi.org/10.1016/j.neuroimage.2011.09.017

Erickson, L. C., Heeg, E., Rauschecker, J. P., & Turkeltaub, P. E. (2014). An ALE meta-analysis on the audiovisual integration of speech signals. *Human Brain Mapping*, *35*(11), 5587-5605. https://doi.org/10.1002/hbm.22572

Gordon, E. M., Laumann, T. O., Marek, S., Newbold, D. J., Hampton, J. M., Seider, N. A., Montez, D. F., Nielsen, A., Van, A. N., & Ortega, M. (2023). A somato-cognitive action network alternates with effector regions in motor cortex. *Nature*, *617*(7960), 351-359. https://doi.org/10.1038/s41586-023-05964-2

Hétu, S., Grégoire, M., Saimpont, A., Coll, M.-P., Eugène, F., Michon, P.-E., & Jackson, P. L. (2013). The neural network of motor imagery : An ALE meta-analysis. *Neuroscience & Biobehavioral Reviews*, *37*(5), 930-949. https://doi.org/10.1016/j.neubiorev.2013.03.017

Hoshi, E., & Tanji, J. (2007). Distinctions between dorsal and ventral premotor areas : Anatomical connectivity and functional properties. *Cognitive neuroscience*, *17*(2), 234-242. https://doi.org/10.1016/j.conb.2007.02.003

Kilteni, K., Andersson, B. J., Houborg, C., & Ehrsson, H. H. (2018). Motor imagery involves predicting the sensory consequences of the imagined movement. *Nature Communications*, *9*(1), 1617.

McDougle, S. D., Tsay, J. S., Pitt, B., King, M., Saban, W., Taylor, J. A., & Ivry, R. B. (2022). Continuous manipulation of mental representations is compromised in cerebellar degeneration. *Brain : A Journal of Neurology*, *145*(12), 4246-4263. https://doi.org/10.1093/brain/awac072

Nachev, P., Kennard, C., & Husain, M. (2008). Functional role of the supplementary and pre-supplementary motor areas. *Nature Reviews Neuroscience*, *9*(11), 856-869. https://doi.org/10.1038/nrn2478

Naito, E., Kochiyama, T., Kitada, R., & others. (2002). Internally simulated movement sensations during motor imagery activate cortical motor areas and the cerebellum. *Journal of Neuroscience*, *22*(9), 3683-3691.

Ramnani, N. (2006). The primate cortico-cerebellar system : Anatomy and function. *Nature Reviews Neuroscience*, *7*(7), 511-522. https://doi.org/10.1038/nrn1953

Rizzolatti, G., & Craighero, L. (2004). The mirror-neuron system. *Annual Review of Neuroscience*, *27*, 169-192.

Rottschy, C., Langner, R., Dogan, I., Reetz, K., Laird, A. R., Schulz, J. B., Fox, P. T., & Eickhoff, S. B. (2012). Modelling neural correlates of working memory : A coordinate-based meta-analysis. *NeuroImage*, *60*(1), 830-846. https://doi.org/10.1016/j.neuroimage.2011.11.050

Turkeltaub, P. E., Eickhoff, S. B., Laird, A. R., Fox, M., Wiener, M., & Fox, P. (2012). Minimizing within-experiment and within-group effects in activation likelihood estimation meta-analyses. *Human Brain Mapping*, *33*(1), 1-13. https://doi.org/10.1002/hbm.21186
